# Unveiling hidden sources of dynamic functional connectome through a novel regularized blind source separation approach

**DOI:** 10.1162/imag_a_00220

**Published:** 2024-07-12

**Authors:** Jialu Ran, Yikai Wang, Ying Guo

**Affiliations:** Department of Biostatistics and Bioinformatics, Emory University, Atlanta, GA, United States

**Keywords:** dynamic functional connectivity, blind source separation, sparse regularization, latent connectivity traits, whole-brain dynamic functional connectivity states, latent sources of functional connectivity

## Abstract

The investigation of the brain’s functional connectome and its dynamic changes can provide valuable insights into brain organization and its reconfiguration. However, the analysis of dynamic functional connectivity (dFC) using functional magnetic resonance imaging (fMRI) faces major challenges, including the high dimensionality of brain networks, unknown latent sources underlying observed dFC, and the large number of brain connections that increase the risk of spurious findings. In this paper, we propose a new regularized blind source separation (BSS) method called dyna-LOCUS to address these challenges. dyna-LOCUS decomposes observed dFC measures to reveal latent source connectivity traits and their dynamic temporal expression profiles. By utilizing low-rank factorization and novel regularizations, dyna-LOCUS achieves efficient and reliable mapping of connectivity traits underlying the dynamic brain functional connectome, characterizes temporal changes of the connectivity traits that contribute to the reconfiguration in the observed dFC, and generates parsimonious and interpretable results in identifying whole-brain dFC states. We introduce a highly efficient iterative Node-Rotation algorithm that solves the nonconvex optimization problem for learning dyna-LOCUS. Simulation studies demonstrate the advantages of our proposed method. Application of dyna-LOCUS to the Philadelphia Neurodevelopmental Cohort (PNC) study unveils latent connectivity traits and key brain connections and regions driving each of these neural circuits, reveals temporal expression levels and interactions of these connectivity traits, and generates new findings regarding gender differences in the neurodevelopment of an executive function-related connectivity trait.

## 1 Introduction

The human brain is a complex network consisting of a large number of functionally linked regions and their connections (Rubinov & Sporns, 2011;Van Den Heuvel & Pol, 2010). The analysis of brain connectomes has emerged as a key area of focus in neuroscience research, providing unprecedented insights into the organization of the brain and its crucial role in neurodevelopment, aging, behavior, as well as the progression and treatment of brain-related diseases (Bullmore & Sporns, 2009;Deco et al., 2011;Kemmer et al., 2018;Satterthwaite et al., 2014;Wang & Guo, 2023;Wang et al., 2016). Functional connectivity (FC) derived from functional imaging such as fMRI has been measured and studied in most neuroimaging studies nowadays. Much of the research on brain connectome analysis has focused on studying stationary or static functional connectivity. Studies have shown that the brain connections undergo dynamic reconfiguration over seconds (Chang & Glover, 2010;Cribben et al., 2012;Ekman et al., 2012), indicating that it is better characterized dynamically. Network reconfigurations may occur when the brain responds to external stimuli, and are potentially even more prominent in the resting state when mental activity is unconstrained (Allen et al., 2014;Leonardi et al., 2014). In recent years, various techniques have been developed to analyze dynamic functional connectivity (dFC). These include the widely used sliding window method (Allen et al., 2014;Chang & Glover, 2010;Kiviniemi et al., 2011;Sakoğlu et al., 2010), as well as other approaches such as Hidden Markov Model (HMM) (Baker et al., 2014;Eavani et al., 2013), dynamic conditional correlations (Lindquist et al., 2014), dynamic connectivity regression (DCR) (Cribben et al., 2012,^2013^), time-frequency approaches (Chang & Glover, 2010;Demirtaş et al., 2016;Yaesoubi et al., 2015), and more. Generally, the dFC methods produce a sequence of symmetric connectivity matrices. Each entry in these matrices corresponds to a connectivity measure, such as the correlation between pairs of brain regions or nodes. These dFC matrices are analyzed to investigate nonstationary changes in brain connectivity over the course of imaging acquisition.

The organization of the brain is a highly intricate system that involves a vast network of neural circuits. The observed dFC matrices reflect collected connectivity patterns across the brain, representing aggregated information contributed by various underlying neural circuits. These underlying circuits can be considered as latent sources or connectivity traits, which represent a collection of connections between different brain regions that tend to occur together during neural processing. The observed dFC data are a combination of these latent connectivity traits. The network reconfigurations in dFC are a result of temporal changes in the expression levels of these latent traits. Gaining a reliable understanding of these latent connectivity traits is crucial for obtaining valuable insights into the architecture and dynamics of brain organization, both in healthy and diseased conditions. For instance, in the field of neurodevelopmental and aging research, studies have demonstrated that different neural circuits undergo maturation at distinct ages during early brain development (Hoff et al., 2013), and they also experience varying rates of deterioration as the brain ages in the elderly population (DeCarli et al., 2012;Iannilli et al., 2017). Additionally, research studies have revealed that demographic or disease-related alterations in the brain connectome typically manifest within specific neural circuits rather than affecting the entire brain connectome as a whole (Mayberg, 2003;Sorg et al., 2007;Williams, 2016). Furthermore, these connectivity traits demonstrate different characteristics. Investigations into dynamic connectivity have uncovered considerable variations in the temporal expression levels of different neurocircuitry subsystems. Certain subsystems demonstrate a higher degree of persistence over time, while others exhibit more transient characteristics (Baker et al., 2014;Chai et al., 2017;Karahanoğlu & Van De Ville, 2015). To gain insights into the brain connectome, it is essential to comprehend the composition and temporal expression profiles of its latent connectivity traits, as well as the ways in which they synchronize and interact with each other. This understanding is critical for unraveling the complex dynamics and functional organization of the brain.

Existing methods for dFC primarily focus on identifying whole-brain dFC states that deviate from stationary FC patterns, without explicitly revealing the underlying connectivity traits and their interplay that give rise to different dFC states (Allen et al., 2014;Karahanoğlu & Van De Ville, 2015;Leonardi et al., 2013). There is a critical need for reliable methods to uncover the latent connectivity traits within dFC and characterize their temporal expression levels, which drive the reconfiguration of dFC patterns. Several significant challenges arise in addressing this need. Firstly, whole-brain dFC matrices are high dimensional, often comprising hundreds of nodes and hundreds of thousands of connections (Chung, 2018;Solo et al., 2018;Wang et al., 2016;G.-R. Wu et al., 2013). This enormous number of connections increases the likelihood of spurious findings when identifying significant edges within the connectivity traits and when examining brain–behavior associations. Moreover, there is a lack of suitable methods to decompose observed dFC matrices and uncover the underlying connectivity traits and their temporal expression levels. Most existing source separation methods, such as Independent Component Analysis (ICA) (Beckmann & Smith, 2004;Calhoun et al., 2001;Shi & Guo, 2016;Wang & Guo, 2019), have mainly focused on decomposing observed neural activity signals such as the blood oxygen level-dependent (BOLD) series from fMRI or the electrodes signal series from EEG. The distinct properties of brain connectivity matrices, in contrast to activity data, limit the applicability of many existing methods. In recent years,Miller et al. (2016)applied tICA, sICA, and PCA methods to decompose dFC data to reveal underlying basis correlation patterns (CPs).Amico et al. (2017)introduced a connectivity-independent component analysis framework (connICA), which vectorizes connectivity matrices and utilizes existing ICA algorithms for decomposition. While the existing ICA, PCA methods, and connICA provide a valuable tool for decomposing connectivity matrices, it has certain limitations that affect the accuracy and reliability of extracting latent connectivity components. For instance, the method treats each connection as an independent sample, disregarding the dependence structure across edges in the brain connectome. This results in a large number of edge-wise parameters for estimation and loss of accuracy. Additionally, without sparsity regularization, the method generates densely connected traits, increasing the likelihood of spurious findings.

In this paper, we propose dyna-LOCUS which is a novel*lo*w-rank decomposition of brain*c*onnectivity with*u*niform*s*parsity for*dyna*mic FC. dyna-LOCUS is a fully data-driven blind source separation method that decomposes dFC matrices to extract latent connectivity traits and characterize their dynamic temporal expression profiles. dyna-LOCUS is a general dFC source separation method that is applicable to connectivity measures derived from various aforementioned dFC techniques. Specifically, dyna-LOCUS decomposes temporally concatenated dFC measures into a combination of latent source signals weighted by temporal mixing coefficients. Each latent source signal corresponds to an underlying connectivity trait and can be mapped back to a connectivity matrix. The mixing coefficients characterize the dynamic temporal expression profile of the connectivity trait. To enhance the accuracy and reliability of recovering the connectivity traits and their temporal profiles, dyna-LOCUS incorporates several innovative and neurobiologically motivated strategies. Firstly, we employ a low-rank structure to model the connectivity traits. This approach is motivated by the observation that brain connectivity trait patterns often exhibit specific structures, such as block-diagonal or banded structures, which can be effectively captured using low-rank matrix factorization techniques (Zhou et al., 2013). By incorporating a low-rank structure, we significantly reduce the number of parameters involved in the estimation process, leading to improved accuracy in extracting the underlying connectivity traits. We then propose a novel angle-based sparsity regularization on the recovered connectivity traits which further increases the reliability of the results by effectively reducing the presence of spurious edges and identifying connections that are genuinely relevant to a specific trait. Furthermore, to improve the accuracy in estimating the temporal expression profiles of the connectivity traits, we propose to include a temporal smoothness regularization in the optimization function. This is motivated by the observation that dynamic FC series typically exhibit a certain degree of temporal coherence, with general patterns displaying continuity across adjacent time windows.

Another major advantage of the dyna-LOCUS is that it provides a highly efficient and convenient approach for brain–behavior modeling and characterization of whole-brain dFC states, which are two major research focuses in dynamic connectome studies. In dyna-LOCUS, the extracted connectivity traits can be viewed as a set of basis connectivity matrices and the mixing coefficients are trait loadings by projecting the observed dFC matrices onto the connectivity trait basis matrices. These trait loadings are essentially low-dimensional representations of the original dFC matrices, characterizing the temporal expression profiles of the connectivity traits. By linking trait loadings with individual demographics, behavioral and clinical measures, we can perform brain–behavior modeling based on dFC to investigate age, gender, or disease-related distinctions in particular dynamic connectome traits. Furthermore, we introduce a novel approach for detecting whole-brain dFC states by employing techniques, such as clustering, on low-dimensional trait loadings. This method is not only computationally efficient but also generates dFC states that are sparser, more reliable, and easier to interpret.

To learn the dyna-LOCUS model, we formulate an optimization function that possesses the desirable property of block multiconvexity. This property ensures the existence of multiple convex subproblems within the optimization framework, facilitating more efficient and reliable parameter estimation. Furthermore, we introduce an efficient node-rotation algorithm, which enhances the effectiveness and computational efficiency of the estimation process. The proposed model and the estimation algorithm demonstrate superior performance in recovering the underlying connectivity traits through extensive simulation studies. We apply dyna-LOCUS to investigate latent connectivity traits underlying resting-state fMRI of the Philadelphia Neurodevelopmental Cohort (PNC) study. dyna-LOCUS successfully identifies latent connectivity traits that exhibit sparse and easily interpretable patterns, many of which demonstrate high reproducibility. Using the temporal mixing coefficients from dyna-LOCUS, we are able to characterize the temporal expression profiles of each of the connectivity traits. This allows us to investigate the dynamic properties of the traits including their energy level and temporal variation and how connectivity traits are synchronized with each other in their dynamics. By employing our novel procedure based on dyna-LOCUS, we successfully identify seven whole-brain dFC states in the PNC study. Notably, our findings exhibit nice agreement with the results obtained using existing whole-brain dFC methods. However, our method and results offer several key advantages including significantly reducing computation time, generating more parsimonious and interpretable whole-brain dFC states, and providing deeper insights into the key latent connectivity traits that drive each state. Furthermore, leveraging results from dyna-LOCUS analysis of the PNC study, we find significant gender differences in the developmental changes in the temporal expression of a connectivity trait driven by the executive function network. This finding introduces new insights to previous research on developmental changes in executive function from childhood to adolescence.

The rest of the paper is organized as follows. InSection 2, the proposed model, estimation, and tuning parameter selection steps are introduced.Section 3illustrates the strength of our method compared with other decomposition methods via simulation studies. InSection 4, we apply the proposed method to investigate dynamic connectivity for the PNC study. Finally, discussions and conclusions are presented inSection 5.

## 2 Materials and Methods

In this section, we introduce dyna-LOCUS, a regularized blind source separation method designed to decompose dynamic functional connectivity (dFC) matrices into a product of latent connectivity traits and temporal mixing matrices. It models the latent traits using a low-rank matrix factorization which is well suited for connectivity matrices. To reduce spurious findings due to large number of edges in brain networks, we develop a novel angle-based element-wise sparsity regularization on the extracted connectivity traits. In addition, we propose a smooth regularization for the temporal mixing time series to account for the similarity in temporally adjacent dynamic connectivity matrices.

### 2.1 dyna-LOCUS model

dyna-LOCUS is applicable to decomposing dynamic FC measures derived from various methods such as the sliding window method (Allen et al., 2014;Hutchison et al., 2013), jackknife correlation (Thompson, Brantefors, et al., 2017;Thompson, Richter, et al., 2017), temporal derivatives (Shine et al., 2015), wavelet coherence (Betzel et al., 2017), dynamic conditional correlation (Lindquist et al., 2014), and more. These methods generate connectivity matrices representing connections between different brain regions at a sequence of time points. For illustration purposes, we present dyna-LOCUS for decomposing connectivity matrices derived from the sliding window approach. The sliding window approach is a widely adopted strategy for investigating dynamic changes in resting-state FC. This technique involves partitioning the scanning time into small windows of fixed duration. These windows can be constructed using either a rectangular window with equal weights or a tapered window that gradually reduces the weights toward the edges. Within each time window, the fMRI BOLD series is utilized to compute the dFC matrix. The window is then shifted in time by a specified number of data points, with partial overlap between consecutive windows. By sliding these windows across the entire scanning session, the method calculates a series of dFC matrices that characterize the time-varying connectivity throughout the scan. Suppose we haveNsubjects, and each subject has fMRI BOLD signal series fromVnodes or regions of interest at$n_{t}$time points. For subjecti, we slide a tapered window (Allen et al., 2014;Rashid et al., 2014) to obtain a series ofV×Vconnectivity matrices denoted as$Y_{i1},Y_{i2},...,Y_{i\;T}$withTbeing the total number of windows.$Y_{it}(t=1,\ldots ,T)$is the connectivity matrix based on thetth sliding window, where$Y_{it}(u,v)\in ℛ$represents the connection between nodesuandvwhich is obtained by a proper transformation of the brain connectivity measure. Since the connectivity matrix$Y_{it}$is symmetric, and the diagonal, which represents self-relationships in the network, is typically not of interest, we define a vector$y_{it}$based on the upper triangular elements of${\text{Y}}_{it}$, i.e.${\text{y}}_{it}=ℒ\left({\text{Y}}_{it}\right)$where$ℒ\left({\text{Y}}_{it}\right)=\left[{\text{Y}}_{it}\left(1,2\right),{\text{Y}}_{it}\left(1,3\right),...,{\text{Y}}_{it}\left(V-1,V\right)\right)]\prime$. Here,$ℒ:{ℛ}^{V\times V}\to {ℛ}^{p}$with$p=\frac{V\times \left(V-1\right)}{2}$.

We propose the following dyna-LOCUS model to decompose the multi-subject dynamic connectivity matrices to extract latent connectivity traits. Specifically, dyna-LOCUS separates the observed connectivity data for theith subject at thetth time window as combinations ofqlatent connectivity sources/traits, that is:

$$y_{it}=\sum_{\ell =1}^{q}a_{it\ell }s_{\ell }+e_{it},$$ (1)

where$s_{\ell }\in {ℛ}^{p}\left(\ell =1,...,q\right)$is the source signal of theℓth latent connectivity source or trait, which is assumed to be independent across theqtraits. A connectivity trait represents a set of brain connections that tend to occur together. The source signal${\text{s}}_{\ell }$includes the weights of each of thepbrain connections in theℓth connectivity trait. By mapping connectivity trait${\text{s}}_{\ell }$back to theV×Vconnectivity matrix form, we can recover the spatial composition of the underlying connectivity pattern.$\left\{a_{it\ell }\right\}$are the mixing coefficients or trait loadings. They represent the presence or prominence of theℓ’s connectivity trait inith subject at time pointt.${\text{e}}_{it}\in {ℛ}^{p}$is an error term independent of source signals. The number of latent sources, i.e.q, can be determined using methods such as the Laplace approximation (Minka, 2000) or based on the reproducibility and interpretability of the extracted latent sources. We can also rewrite the model (1) in the following matrix form aggregating across time windows and subjects:

Y=AS+E, (2)

where$\text{Y}={\left[{\text{y}}_{11},\ldots ,{\text{y}}_{1T},\ldots ,{\text{y}}_{N1},\ldots ,{\text{y}}_{NT}\right]}^{\prime }\in {ℛ}^{NT\times p}$is the multi-subject dynamic connectivity data,$\text{S}=[{\text{s}}_{1},\ldots ,{\text{s}}_{q}{]}^{\prime }$$\in {ℛ}^{q\times p}$is the connectivity trait matrix,$\text{A}=\left\{a_{it\ell }\right\}\in {ℛ}^{NT\times q}$is the mixing or trait loading matrix, and$\text{E}=[{\text{e}}_{11},\ldots ,{\text{e}}_{1T},\ldots ,$${\text{e}}_{N1},\ldots ,{\text{e}}_{NT}{]}^{\prime }\in {ℛ}^{NT\times p}$.

Motivated by the observations that brain connectivity matrices often have block or banded structures, we model connectivity traits with a low-rank structure which can efficiently capture such kind of characteristics (Zhou et al., 2013) using a much smaller number of parameters. Specifically,

$${\text{s}}_{\ell }=ℒ\left({\text{X}}_{\ell }{\text{D}}_{\ell }{{\text{X}}^{\prime }}_{\ell }\right)=ℒ\left(\sum_{r=1}^{R_{\ell }}d_{\ell }^{\left(r\right)}{\text{x}}_{\ell }^{\left(r\right)}{\text{x}}_{\ell }^{{\left(r\right)}^{\prime }}\right),\ell \in 1,\;2,...,q.$$ (3)

Here, the source signal${\text{s}}_{\ell }$is modeled via a low-rank factorization, where${\text{X}}_{\ell }=\left[{\text{x}}_{\ell }^{\left(1\right)},\ldots ,{\text{x}}_{\ell }^{\left(R_{\ell }\right)}\right]\in {ℛ}^{V\times R_{\ell }}$with rank$R_{\ell }<V$and each tensor factor${\text{x}}_{\ell }^{\left(r\right)}$($r=1,\ldots ,R_{\ell }$) is aV×1vector with unit norm, i.e.$||{\text{x}}_{\ell }^{\left(r\right)}|{|}_{2}\;=1$for identifiability purpose.${\text{D}}_{\ell }$is a diagonal matrix with diagonal elements${\text{d}}_{\ell }=\left(d_{\ell }^{\left(1\right)},..,d_{\ell }^{\left(R_{\ell }\right)}\right)$. The low-rank factorization implies theVnodes reside in a reduced subspace with the dimension of$R_{\ell }$, i.e.${\text{s}}_{\ell }=ℒ\left(\sum_{r=1}^{R_{\ell }}d_{\ell }^{\left(r\right)}{\text{x}}_{\ell }^{\left(r\right)}{\text{x}}_{\ell }^{{\left(r\right)}^{\prime }}\right),$where therth tensor factor${\text{x}}_{\ell }^{\left(r\right)}\in {ℛ}^{V\times 1}$represents the latent coordinates of theVnodes in therth dimension and each row of${\text{X}}_{\ell }$, i.e.${\text{x}}_{\ell }(v)\in {ℛ}^{R_{\ell }\times 1}$, represents the latent coordinates of thevth node in the$R_{\ell }$-dimensional latent subspace.$d_{\ell }^{\left(r\right)}$reflects the contribution of therth dimension in generating${\text{s}}_{\ell }$. Note that we specify trait-specific rank$R_{\ell }$in our low-rank model (3) which provides the flexibility to accommodate various connectivity traits with different network properties and topological structures.

The proposed low-rank model offers several advantages for modeling the brain connectome. Firstly, by incorporating a low-rank structure, the model achieves a substantial reduction in the number of parameters required to represent the brain connections. The number of parameters decreases from a quadratic complexity of$O(V^{2})$to a linear complexity ofO(V). This reduction in parameter space improves computational efficiency and alleviates the risk of overfitting, particularly in scenarios where the number of brain regions (V) is large. Secondly, the low-rank factorization offers appealing neuroscience interpretations in modeling brain connectivity. In the low-rank model, a nodevis characterized by its latent coordinates in the$R_{\ell }$-dimensional subspace, i.e.${\text{x}}_{\ell }\left(v\right)$, which potentially reflect the node’s underlying neural activity. The connection between two nodes in theℓth trait is modeled as${\text{S}}_{\ell }\left(u,v\right)={\text{x}}_{\ell }{\left(u\right)}^{\prime }{\text{D}}_{\ell }{\text{x}}_{\ell }\left(v\right)$, which is the inner product between their latent coordinates. This implies the connection between the nodes depends on the similarity between the neural activity of the nodes characterized by their latent coordinates, which aligns with the understanding of brain connectivity in neuroscience (Friston, 2011;Friston et al., 1993). Another desirable property of this model is that all the brain connections involving a nodevare based on the node’s latent coordinate${\text{x}}_{\ell }(v)$and hence are inherently related. This appropriately accounts for the dependence structure across edges in the brain connectome, which is disregarded by many existing methods. Finally, the latent coordinate${\text{x}}_{\ell }(v)$can help identify key nodes driving each connectivity trait. To this end, we propose the following node contribution index to characterize a node’s contribution to a connectivity trait,

$$g_{\ell }\left(v\right)={\left\|{\tilde{\text{D}}}_{\ell }^{\frac{1}{2}}{\text{x}}_{\ell }\left(v\right)\right\|}_{2}^{2},$$ (4)

where${\tilde{\text{D}}}_{\ell }$is a scaling matrix based on${\text{D}}_{\ell }$that scales nodev’s$R_{\ell }$-dimensional latent coordinate${\text{x}}_{\ell }(v)$with the dimensions’ contribution in generating the source signals. The index$g_{\ell }(v)$measures the scaled magnitude of the nodevin the latent subspace of the connectivity trait and reflects the contribution of this node to generating the connectivity trait. The node contribution index can be used to identify key nodes driving each connectivity trait.

We present a schematic plot of the proposed dyna-LOCUS model inFigure 1. The model decomposes observed dFC measures to generate two key results: (1) the source signals$\left\{{\text{s}}_{\ell }\right\}$that can be mapped back to the brain to depict the spatial composition of the connectivity traits and (2) the trait loadings$\left\{a_{it\ell }\right\}$that characterize the temporal expression profiles of the connectivity traits. The information provided by the dyna-LOCUS model offers valuable information that can assist in understanding the intricate dynamics and underlying organization of the brain connectome.

**Fig. 1.**
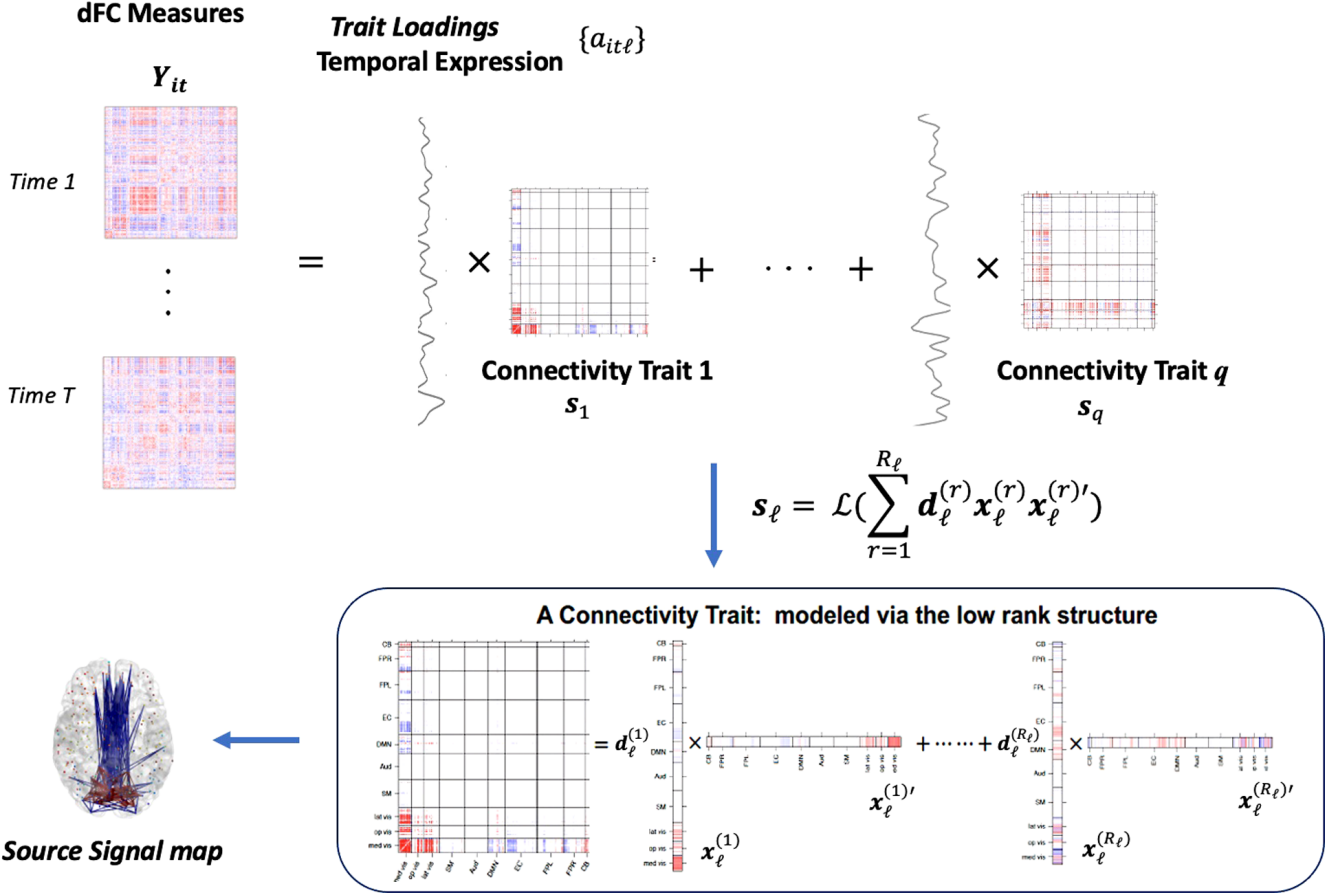
Schematic plot of dyna-LOCUS.

### 2.2 Regularizations in dyna-LOCUS learning

To enhance the reliability in mapping dynamic functional connectivity traits, we integrate regularizations in dyna-LOCUS learning. These include a sparsity regularization for recovering the spatial source maps of the connectivity traits to reduce spurious connections and a temporal smoothness regularization for recovering the temporal expression profiles of the connectivity traits. The motivation for the spatial sparsity regularization derives from existing findings that functional connections in neural circuits exist only in a proportion of region pairs across the brain (Bullmore & Sporns, 2009;Huang et al., 2010;Lee et al., 2011). Given the enormous number of region pairs in a connectivity matrix, appropriate sparsity control is needed in order to obtain parsimonious results in recovering the spatial source maps of the connectivity traits. To this end, we propose a novel sparsity method that aims to achieve element-wise sparsity on the reconstructed connectivity traits based on the low-rank structure. Another observation from previous studies (Monti et al., 2017;Zhang et al., 2021) and our experiments is that the overall pattern of dynamic functional connectivity matrices generally demonstrates temporal similarity between adjacent time windows. To account for this, we include a temporal smoothness regularization on the trait loading series.

With the regularizations, the proposed dyna-LOCUS model is learned via the following optimization,

$$\begin{array}{l} \text{min}\sum_{i=1}^{N}\overset{T}{\underset{t=1}{\;\sum }}||{\text{y}}_{it}-\sum_{\ell =1}^{q}a_{it\ell }ℒ\left({\text{X}}_{\ell }{\text{D}}_{\ell }{{\text{X}}^{\prime }}_{\ell }\right){||}_{2}^{2}\\ \;\;\;\;\;+\phi \sum_{\ell =1}^{q}\;\sum_{u<v}|{\text{x}}_{\ell }{\left(u\right)}^{\prime }{\text{D}}_{\ell }{\text{x}}_{\ell }\left(v\right)|+\lambda \sum_{i=1}^{N}\;\sum_{t=2}^{T}||{\text{a}}_{it}-{\text{a}}_{i\left(t-1\right)}{||}_{2}^{2}, \end{array}$$ (5)

whereϕis the tuning parameter for the sparsity control andλis the tuning parameter for the temporal smoothness regularization,$a_{it}\in {ℛ}^{q}$is thetth row of theith participant’s trait loading matrix representing theith participant’s loadings on theqconnectivity traits at timet. The first term in the optimization function (5) measures the closeness between the observed dFC and the reconstructed dFC based on the dyna-LOCUS model. The second term is the sparsity regularization aiming to achieve element-wise sparsity on the connectivity traits modeled via the low-rank structure, i.e.$S_{\ell }(u,v)=x_{\ell }{\left(u\right)}^{'}D_{\ell }x_{\ell }(v).$. The sparsity penalty term in our model aims to minimize the sum of inner products of the latent coordinates for all pairs of nodes in the brain. This inner product corresponds to the angle between two nodes in the latent subspace. Consequently, our sparsity penalization can be understood as an angle-based regularization. By using an angle-based sparsity regularization, we are able to induce sparsity in the dependencies between pairs of nodes. This approach is both intuitive and theoretically sound, because our objective is to achieve sparsity in the connections between nodes. It is worth noting that in addition to the L1 norm specified in (5), alternative penalization functions such as SCAD (Fan & Li, 2001) can also be adopted for our sparsity regularization. The third term in (5) is the regularization for temporal smoothness in the mixing matrix and is based on the differences in the temporal mixing coefficients at adjacent time points.

Prior to the decomposition, several preprocessing steps, including centering, dimension reduction, and whitening, which are commonly adopted in blind source separation, are applied to the multi-subject connectivity dataY. These preprocessing steps facilitate the subsequent decomposition by reducing the computational load and avoiding overfitting (Hyvärinen et al., 2001). Following the preprocessing procedures of the previous work (Beckmann & Smith, 2004;Shi & Guo, 2016;Wang & Guo, 2023), we first demeanYand then perform the dimension reduction and whitening as$\tilde{\text{Y}}=\text{HY}$withHbeing the whitening matrix. The detailed description ofHis available inAppendix A. The preprocessed data$\tilde{\text{Y}}$is of dimensionq×p,where the columns correspond topconnections in the brain.

With the preprocessing, the dyna-LOCUS model in (2) can be rewritten on the reduced and sphered space as follows:

$$\tilde{\text{Y}}=\tilde{\text{A}}\text{S}+\tilde{\text{E}},$$ (6)

where$\tilde{\text{A}}=\text{HA}=\left\{{\tilde{a}}_{i\ell }\right\}\in {ℛ}^{q\times q}$,$\tilde{\text{E}}=\text{HE}$. Note that the dimension reduction in the preprocessing is performed on the row space ofY,which corresponds to the subject and time domain, and does not affect the column space ofY,which corresponds to the connectivity domain. Therefore, the connectivity traitSis unaffected by the preprocessing. As in previous blind source separation methods (Beckmann & Smith, 2004;Hyvärinen & Oja, 2000), the whitening in the preprocessing leads to an orthogonal mixing matrix on the reduced space$\tilde{\text{A}}$which facilitates the subsequent model estimation. With the preprocessed data and the orthogonal mixing matrix, the final optimization function for dyna-LOCUS is

$$\begin{array}{l} {\text{min}}_{\tilde{\text{A}},\left\{{\text{X}}_{\ell },{\text{D}}_{\ell }\right\}}\sum_{l=1}^{q}||{\tilde{\text{Y}}}^{\prime }{\tilde{\text{a}}}_{\ell }-ℒ\left({\text{X}}_{\ell }{\text{D}}_{\ell }{{\text{X}}^{\prime }}_{\ell }\right){||}_{2}^{2}\\ \;\;\;\;+\;\phi \overset{q}{\underset{\ell =1}{\sum \;}}\sum_{u<v}|{\text{x}}_{\ell }{\left(u\right)}^{\prime }{\text{D}}_{\ell }{\text{x}}_{\ell }\left(v\right)|+\lambda ||\text{W}\tilde{\text{A}}{||}_{F}^{2}, \end{array}$$ (7)

where${\tilde{\text{a}}}_{\ell }$is theℓth column of$\tilde{\text{A}}$,${\text{H}}^{*}$is the dewhitening matrix, and$\text{W}\;={\text{R}}^{*}{\text{H}}^{*},$where${\text{R}}^{*}$is a temporal contrasting matrix that measures the differences in the temporal mixing coefficients at adjacent time points. The closed forms of the whitening matrixHand the dewhitening matrix${\text{H}}^{*}$, as well as the derivation of the final optimization function (7), are provided inAppendix A.

### 2.3 Estimation algorithm and tuning parameters selection

The objective function in (7) is nonconvex but can be shown to be block multiconvex. A function is block multiconvex if there exists a partition of the set of parameters satisfying that the function is convex with respect to each of the individual arguments in the partition, while holding the others fixed. We can show the optimization function (7) is block multiconvex with respect to the partition of$P=\{x_{1}(1),\ldots ,x_{1}(V),\ldots ,x_{q}(V),d_{1},\ldots ,d_{q},\tilde{A}\}.$Based on the block multiconvexity property, we derive an efficient node-rotation learning algorithm with closed-form solutions in each updating step. We initialize the algorithm with${\hat{\tilde{\text{A}}}}^{\left(0\right)},\left\{{\hat{\text{X}}}_{\ell }^{\left(0\right)},{\hat{\text{D}}}_{\ell }^{\left(0\right)}\right\}$derived from estimates based on existing methods such as connICA. The algorithm then estimates the parameters by iterating the following updating steps:*Step 1: Updating*${\text{X}}_{\ell }$. FollowingWang and Guo (2023), we develop a node-rotation algorithm that updates${\text{X}}_{\ell }$at one of the nodevwhile conditioning on the rest of the nodes and then rotating across the nodes. Specifically, at thekth iteration, we update${\hat{\text{x}}}_{\ell }^{\left(k\right)}\left(v\right)$,v=1,..,V, conditioning on${\hat{\tilde{A}}}^{\left(k-1\right)}$,${\hat{D}}_{\ell }^{\left(k-\right)}$and${\hat{\text{X}}}_{\ell }{\left(-v\right)}^{\left(k-1\right)}$.*Step 2: Updating*${\text{D}}_{\ell }$. The second step updates the diagonal estimates of the diagonal of${\text{D}}_{\ell }$, i.e.${\text{d}}_{\ell },\ell =1,...,q$, conditioning on the estimates of${\hat{\text{X}}}_{\ell }^{\left(k\right)}$and${\hat{\tilde{\text{A}}}}^{\left(k-1\right)}$.*Step 3: Updating*$\tilde{\text{A}}$. An advantage of our proposed temporal smoothness regularization is that it admits an analytical form of solution to the temporal mixing matrix$\tilde{\text{A}}$. With that, we obtain the updated mixing matrix${\hat{\tilde{\text{A}}}}^{\left(k\right)}$based on the estimates of$\left\{{\hat{\text{X}}}_{\ell }^{\left(k\right)},{\hat{\text{D}}}_{\ell }^{\left(k\right)}\right\}$. The details of the node-rotation estimation algorithm are provided inAppendix B. Developed based on the block multiconvexity of the objective function, the node-rotation algorithm has the appealing theoretical property that updating each block of the parameters can be performed via convex optimization. Though being a highly efficient algorithm with analytic solutions, the node-rotation algorithm does involve rotating across the nodes to update the latent coordinates of each node. To further increase computation efficiency in learning dyna-LOCUS, we also develop an alternative estimation algorithm that simultaneously updates the latent coordinates of all the nodes using an eigenvalue decomposition. This alternative estimation algorithm could help reduce computation time for studies with large sample sizes and brain atlases involving a large number of nodes. Details on the alternative algorithm are presented in Section 7 of theSupplementary Material.

The optimization function in (7) involves three sets of tuning parameters: the rank parameters${\left\{R_{\ell }\right\}}_{\ell =1}^{q}$that control the dimension of the subspace of the connectivity traits,ϕwhich regulates the influence of the sparsity penalization for the connectivity traits, andλwhich regulates the influence of the temporal smoothness in the mixing matrix. In the dyna-LOCUS model, we specify trait-specific rank parameters${\left\{R_{\ell }\right\}}_{\ell =1}^{q}$in the low-rank factorization in order to accommodate the difference in the topology and structure across connectivity traits. However, selecting the appropriate rank parameter for each trait can be challenging using conventional approaches. To address this challenge, we propose an adaptive selection approach (Wang & Guo, 2023) to efficiently choose$\left\{R_{\ell }\right\}$for theqlatent sources. One of the main objectives of incorporating a low-rank structure is to utilize fewer parameters while effectively capturing the connectivity source signals. Therefore, our approach chooses the rank parameter$R_{\ell }$to achieve a desired level of similarity between the estimated source signals with the low-rank structure, i.e.${\hat{\text{s}}}_{\ell }$, and the unconstrained source signal estimate obtained without assuming the low-rank structure, which is denoted as${\hat{\text{s}}}_{\ell }^{*}$. The unconstrained estimate${\hat{\text{s}}}_{\ell }^{*}$can be conveniently obtained from our algorithm as an intermediate result before we project the estimates onto the reduced subspace spanned by the low-rank structure. Specifically,$R_{\ell }$is selected to be the smallest integer value such that,

$${\left\|{\hat{\text{s}}}_{\ell }-{\hat{\text{s}}}_{\ell }^{*}\right\|}_{2}^{2}/{\left\|{\hat{\text{s}}}_{\ell }^{*}\right\|}_{2}^{2}\leq 1-\rho ,$$ (8)

whereρ∈(0,1)is a proportion parameter controlling the desired level of similarity between the unconstrained and low-rank structured estimates for the latent sources. Once the proportion parameterρis specified, the proposed approach adaptively selects the rank for each of the latent sources. The proposed method not only allows us to adaptively select an appropriate rank parameter to capture the varying patterns of each connectivity trait but also considerably simplifies the challenging task of selectingqrank parameters to only select a single parameterρ. With the proposed adaptive selection approach, we propose to selectρandϕ, which are parameters related to learning the latent connectivity sources, via a BIC-type criterion,

$$\text{BIC}=-2\sum_{i=1}^{q}\;log\left(g\left({\tilde{\text{y}}}_{\text{i}};\sum_{j=1}^{q}\;{\hat{\tilde{a}}}_{ij}{\hat{\text{s}}}_{j};\hat{\tilde{\sigma }}\;\;{\text{I}}_{p}\right)\right)+log\left(N\right)\sum_{j=1}^{q}{\left\|{\hat{\text{s}}}_{j}\right\|}_{0},$$ (9)

where${\tilde{\text{y}}}_{\text{i}},i=1,...,q$is theith row in$\tilde{\text{Y}}$,$\left\{{\hat{\tilde{a}}}_{ij}\right\}$are the estimated mixing coefficients of preprocessed connectivity data,${\hat{\text{s}}}_{j},j=1,...,\ell$, is the estimated connectivity trait,${\hat{\tilde{\sigma }}}^{2}=\frac{1}{qp}\sum_{i=1}^{q}{\left\|{\tilde{\text{y}}}_{\text{i}}-\sum_{i=1}^{q}{\hat{\tilde{a}}}_{ij}{\hat{\text{s}}}_{j}\right\|}_{2}^{2}$,gis the pdf of a multivariate Gaussian distribution, and$||.{||}_{0}$denotes the$L_{0}$norm which evaluates the number of nonzero elements in a vector. The BIC criterion seeks to achieve a balance by maximizing the likelihood of the observed dFC data while simultaneously minimizing the nonzero elements in the latent connectivity sources for the purpose of sparsity. The BIC criterion serves as a valuable guide in selecting the tuning parametersϕandρ. However, it is worth noting that the choice may not always be straightforward solely based on BIC in practice. Therefore, besides the BIC criterion, users can also employ supplementary selection strategies, such as specifying tuning parameters based on the desired sparsity level and the neuroscience interpretations they aim to achieve in the extracted connectivity traits. After obtainingϕandρ, we selectλbased on the goodness-of-fit of the model by computing the mean-squared reconstruction error, i.e.$||\text{Y}-\hat{\text{A}}\hat{\text{S}}|{|}_{F}^{2}$.

### 2.4 Reproducibility/reliability of the extracted connectivity traits

One important criterion for evaluating the connectivity traits extracted from the imaging data is the reproducibility of the traits across replicated samples which reflects the reliability of the traits (Amico et al., 2017;Wang & Guo, 2023). To this end, we assess the reproducibility of the connectivity traits extracted by dyna-LOCUS from the PNC study across replicated bootstrap data samples using the following reliability index for blind source separation methods (Kemmer et al., 2018),

$$RI_{\ell }=\frac{\frac{1}{B}\sum_{b=1}^{B}\left\{h\left({\hat{\text{s}}}_{\ell },{\hat{\text{s}}}_{\ell }^{\left(b\right)}\right)\right\}-\frac{1}{Bq}\sum_{b=1}^{B}\sum_{j=1}^{q}\left\{h\left({\hat{\text{s}}}_{\ell },{\hat{\text{s}}}_{j}^{\left(b\right)}\right)\right\}}{1-\frac{1}{B}\sum_{b=1}^{B}\left\{h\left({\hat{\text{s}}}_{\ell },{\hat{\text{s}}}_{\ell }^{\left(b\right)}\right)\right\}},$$ (10)

wherehis a similarity measure such as the correlation coefficient or Jaccard Index,Bis the total number of replicated samples (i.e. bootstrap data samples),${\hat{\text{s}}}_{\ell }$is the latent sources extracted from the original data, and${\hat{\text{s}}}_{\ell }^{\left(b\right)}$is the latent source estimated from thebth bootstrap data sample that is matched with${\hat{\text{s}}}_{\ell }$from the original data. Following previous work (Keeratimahat & Nichols, 2022;B. Wu et al., 2022), we employ a greedy matching algorithm to match the latent sources from the bootstrap sample and the original data.

The reliability index$RI_{\ell }\left(\ell =1,\ldots ,q\right)$provides a scaled and chance-corrected measure to assess the reproducibility of each latent source. The index reflects the similarity of an extracted latent source from the original data and its matching estimates across the replication samples, removing by-chance similarity between the original latent source and any of theqextracted latent sources estimates. It is further scaled by its maximum possible value so that it typically ranges from 0 to 1, where$RI_{\ell }$= 0 indicates theℓth latent source is not reproducible across replication samples after correcting for by-chance similarity and$RI_{\ell }$close to 1 indicates that the latent source is highly reproducible across replication samples. The reliability index is formulated in a similar way as the Cohen’s kappa coefficient (Cohen, 1960). We can follow the kappa’s guideline to interpret the reliability index. That is, we interpret$RI_{\ell }\leq 0$as none reproducibility, 0.01–0.20 as slight, 0.21–0.40 as fair, 0.41–0.60 as moderate, 0.61–0.80 as substantial, and 0.81–1.00 as almost perfect reproducibility. As a scaled and chance-corrected measure,$RI_{\ell }$is comparable across different latent sources, i.e. connectivity traits, making it a desirable reliability measure for blind source separation methods (Kemmer et al., 2018;Wang & Guo, 2023).

### 2.5 Investigating brain dynamic connectome using dyna-LOCUS

In this section, we present several analysis strategies that leverage the results obtained from dyna-LOCUS to provide a detailed understanding of the complex dynamics of the brain’s functional connectome. These include understanding the temporal expression variations and properties of the connectivity traits, exploring the interactions between different connectivity traits, and identifying and characterizing whole-brain dFC states.

#### 2.5.1 Temporal expression of the latent connectivity traits

A valuable output from dyna-LOCUS is individual-level time-dependent loadings of each connectivity trait. These trait loadings contain information on the temporal expression of each of the latent connectivity traits in an individual’s brain connectivity. We explore the dynamics of latent connectivity traits by examining two key aspects: the energy and variation of their temporal expression. Additionally, we investigate whether and how the connectivity traits synchronize with each other by studying the associations between their temporal loadings.

To identify highly expressed connectivity traits, we quantify the overall dynamic activation across the time windows of a connectivity trait expression by evaluating the energy of the temporal loading of the trait over the scanning session for each individual. Specifically, for individuali, the energy of connectivity traitℓis defined as$\sum_{t=1}^{T}a_{it\ell }^{2}$. Furthermore, to assess how stable or transient a connectivity trait is, we quantify the variation of the temporal expression of a connectivity trait by evaluating the mean of the absolute relative change of trait loadings throughout the scanning session. In particular, for individuali, the variation of connectivity traitℓis defined as$\sum_{t=1}^{T-1}\frac{1}{T-1}\left|\frac{a_{i\left(t+1\right)\ell }-a_{it\ell }}{a_{it\ell }}\right|$.

To investigate the interaction and synchronization between connectivity traits, we propose to evaluate the cross-correlation function (CCF) between trait loading time series. Denote$\left\{x_{t}\right\},\left\{y_{t}\right\},\left(t=1,\ldots ,T\right)$as the trait loading time series of two connectivity traits of an individual. To facilitate notation, we order them by denoting the trait with higher reproducibility, i.e. higher RI index, as$x_{t}$and the trait with lower reproducibility as$y_{t}$. For a pair of traits, the CCF with a lag ofkis${\text{CCF}}_{k}\left(x_{t},y_{t}\right)=\frac{\frac{1}{n}\sum_{t=1}^{n-k}\left(x_{t+k}-\bar{x}\right)\left(y_{t}-\bar{y}\right)}{SD_{x}SD_{y}}$, and CCF with a lag of−kis${\text{CCF}}_{-k}\left(x_{t},y_{t}\right)=\frac{\frac{1}{n}\sum_{t=1}^{n-k}\left(x_{t}-\bar{x}\right)\left(y_{t+k}-\bar{y}\right)}{SD_{x}SD_{y}}$, where$\bar{x}$and$\bar{y}$are the mean value of$\left\{x_{t}\right\}$and$\left\{y_{t}\right\}$respectively, and$SD_{x}$and$SD_{y}$are their standard deviations. A large magnitude of CCF between a pair of connectivity traits indicates synchronized temporal expression levels either in the same or in opposite direction. For each pair of connectivity traits, we identify the lag on the population level when they achieve the highest level of synchronization by taking the mode of the lags when the highest synchronization is achieved among individuals.

#### 2.5.2 Identifying whole-brain dFC states

Another major contribution of dyna-LOCUS is that it provides a highly efficient and reliable approach for the identification and characterization of whole-brain dynamic connectivity states. In current neuroimaging studies, whole-brain dFC states are often obtained by clustering the observed dFC matrices (Allen et al., 2014;Damaraju et al., 2014;De Lacy et al., 2017;Geng et al., 2020). Using dyna-LOCUS, we propose a new approach by conducting the analysis on subjects’ trait loadings, which are low dimensional representations of the observed dFC matrices, and then obtain the whole-brain dFC states via reconstruction. This method not only dramatically improves the computational efficiency but also generates dFC states that are sparser, more reliable, and easier to interpret.

We illustrate our new pipeline for investigating whole-brain dFC states inFigure 2. First, we apply dyna-LOCUS to decompose the observed dFC matrices to extract connectivity traits and individual-level trait loading time series. Here, connectivity traits can be viewed as a set of basis matrices for observed dFC data, and the trait loadings are low-dimensional representations of the observed dFC matrices obtained by projecting them onto the basis. Therefore, rather than clustering the dFC matrices to identify dFC states, we can cluster the low-dimensional trait loadings to identify cluster centroids, i.e. cluster medians. The whole-brain dFC states can then be reconstructed by multiplying the centroid trait loadings with the connectivity trait basis matrices.

**Fig. 2.**
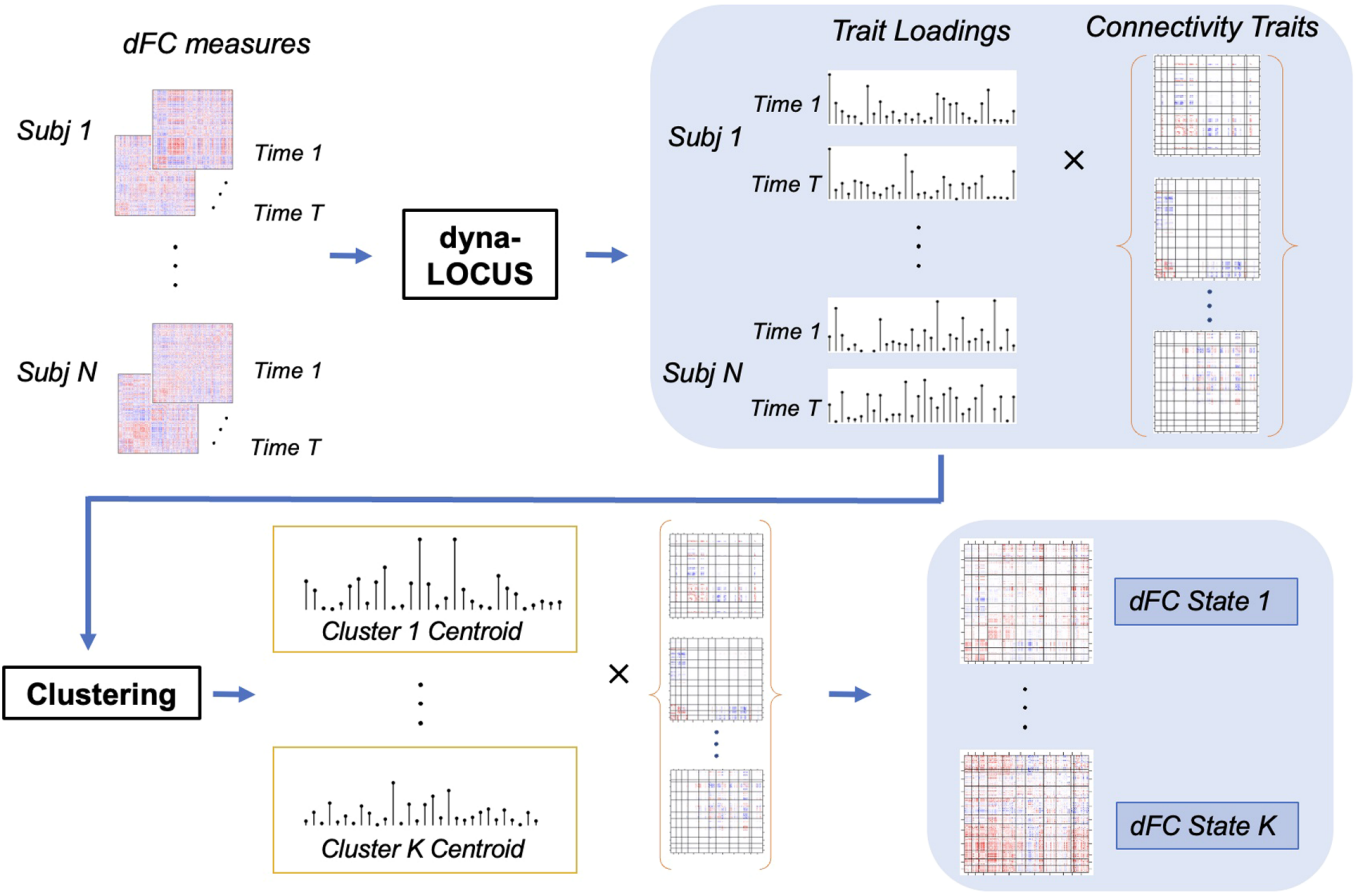
Schematic plot of the pipeline for identifying whole-brain dFC states based on dyna-LOCUS.

## 3 Simulation

In this section, we investigate the performance of our model based on simulation studies. We compare the performance of dyna-LOCUS with two other source separation methods: connICA (Amico et al., 2017), which is a recently developed connectivity ICA method, and the dictionary learning (DL) method (Mairal et al., 2009), which is a popular sparse decomposition method. DL minimizes the$L_{1}$norm of${\text{s}}_{\ell }$, and aims to achieve sparse estimates for the connectivity traits.

We specifyV=50, q=8, consider two sample sizesN=20,50andT=36windows. We generate the eight latent connectivity source signals based on specific connectivity patterns that we observe from the connectivity traits extracted from real imaging data including the PNC study. The mixing coefficients are also sampled from estimates from real imaging data. Furthermore, we add zero mean Gaussian noises to the mixture of signals where the variance is specified based on the signal-to-noise ratio observed from real data. Specifically, we consider three variance settings with$\sigma^{2}=0.5^{2},1.5^{2}$, and$2.5^{2}$, corresponding to low, medium, and high variance levels, respectively. In summary, we have2×3simulation settings with combinations of sample sizes and variance levels. For each setting, we generate100simulation runs to capture the variations in performance. Based on BIC and the goodness-of-fit criteria, we selected the following tuning parameters:ρ=0.95,λ=0.01, andϕ=1.5.

Following previous work (Beckmann et al., 2005;Wang & Guo, 2019,^2023^), we evaluate the performance of each method based on the correlations between the truth and the model-based estimates on the source signals and mixing coefficients. We further examine the standard deviation of the correlations across100simulation runs to evaluate the robustness of the methods.

**Fig. 3.**
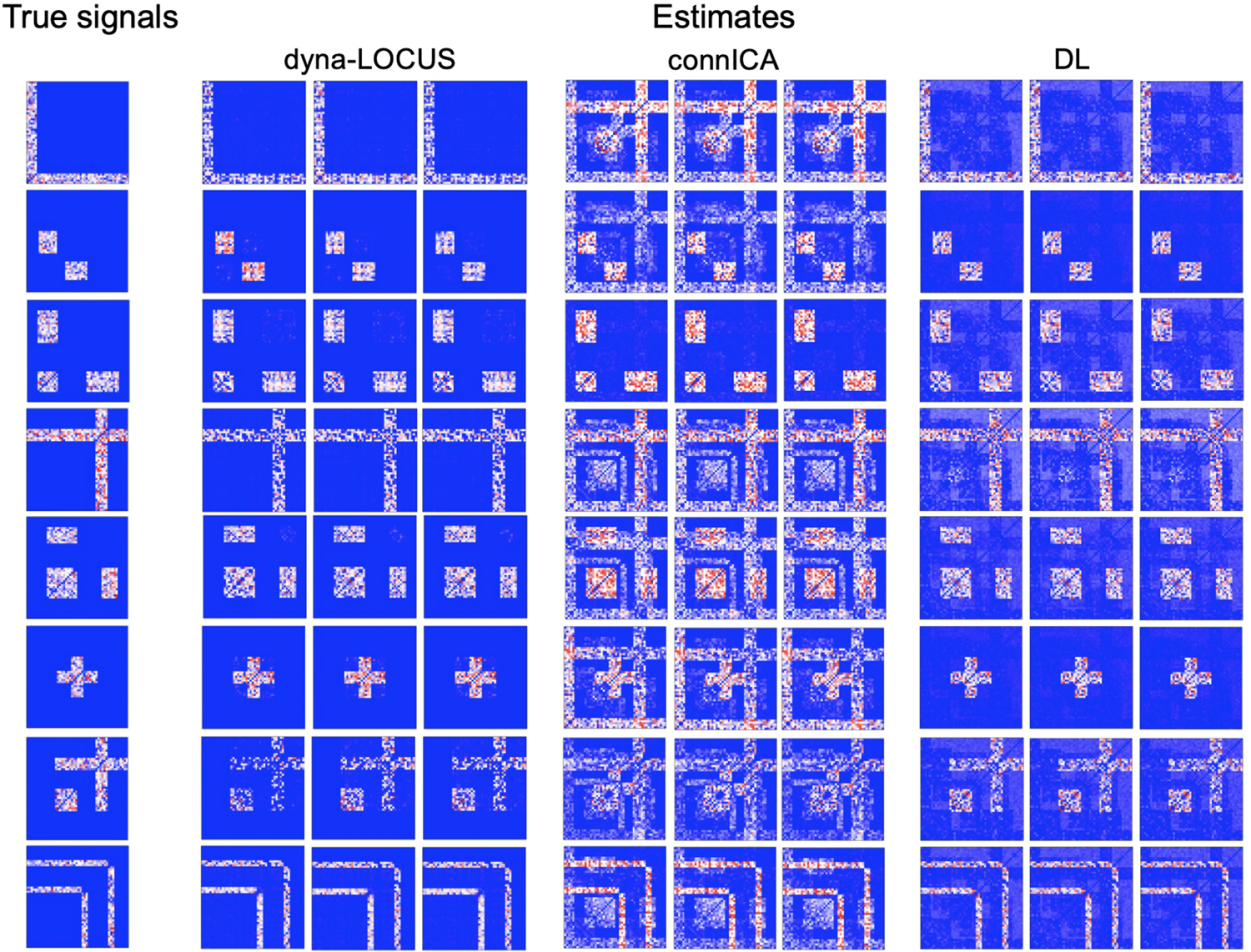
Results from the simulation study. The figures illustrate the true source signals and the estimated signals by dyna-LOCUS, connICA, and dictionary learning (DL) in three randomly selected simulation runs conducted under the low-level variance setting.

Results are summarized inTable 1andFigure 3.Table 1shows dyna-LOCUS consistently demonstrates better accuracy in recovering the latent sources and mixing coefficients as compared with connICA and DL. The standard deviation of dyna-LOCUS is generally lower than that of the other two methods, indicating our proposed method has better stability.Figure 3shows that dyna-LOCUS demonstrates considerably better performance in recovering the spatial compositions of the connectivity traits. Compared with the results by the two existing methods, the source signal maps by dyna-LOCUS are more accurate, sparser with much fewer false positive findings, and show little crossing talking problems. Specifically, connICA, being a decomposition method without sparsity constraints and the low-rank structure, tends to yield noisy and inaccurate estimates. As a sparse decomposition method, DL minimizes the$L_{1}$norm of${\text{s}}_{\ell }$to achieve sparse estimates for the source signals. However, it does not model the source signals using the low-rank structure, which disregards the interdependence among brain connections. Instead, it treats connections as independent parameters, leading to a large number of parameters for DL to learn. As a result, DL estimations may lack accuracy compared with the proposed dyna-LOCUS approach, as evidenced by simulation results.

**Table 1.** Simulation results for comparing dyna-LOCUS and the existing connICA and DL methods based on 100 simulation runs conducted under three variance (Var.) settings.

Term	N	Var.	dyna-LOCUS	connICA	DL
Latent source corr. (SD)	20	Low	0.955 (0.005)	0.805 (0.018)	0.947 (0.000)
		Mid	0.943 (0.005)	0.749 (0.014)	0.882 (0.017)
		High	0.828 (0.015)	0.626 (0.019)	0.772 (0.016)
	50	Low	0.956 (0.004)	0.784 (0.005)	0.955 (0.000)
		Mid	0.955 (0.005)	0.763 (0.007)	0.927 (0.001)
		High	0.932 (0.005)	0.716 (0.004)	0.884 (0.002)
Loading matrix corr. (SD)	20	Low	0.988 (0.002)	0.801 (0.011)	0.962 (0.000)
		Mid	0.950 (0.002)	0.771 (0.010)	0.930 (0.012)
		High	0.839 (0.011)	0.684 (0.018)	0.848 (0.020)
	50	Low	0.989 (0.001)	0.790 (0.005)	0.960 (0.000)
		Mid	0.956 (0.002)	0.764 (0.007)	0.928 (0.001)
		High	0.885 (0.003)	0.710 (0.004)	0.870 (0.002)

Values presented are mean and standard deviation of correlations between the true and estimated latent sources and loading/mixing matrices.

In addition to the simulation results presented inTable 1andFigure 3, we further consider two additional scenarios with decreasing levels of sparsity and an increased number of connections in the true source signals. These additional simulations aim to evaluate the performance of dyna-LOCUS across varying degrees of source signal sparsity. Across the different sparsity levels, dyna-LOCUS consistently exhibits superior accuracy in recovering the underlying source signals and their respective temporal loadings compared with the other two methods. Please refer to Section 4 of theSupplementary Materialfor the additional simulation studies. In Supplementary Material Section 5, we present another simulation study in which we simulate dFC data using true source signals derived from connectivity traits extracted from the PNC study. This simulation demonstrates the advantages of dyna-LOCUS compared to connICA and DL as well.

## 4 Investigating Dynamic Functional Connectome for the Philadelphia Neurodevelopmental Cohort (PNC) Study

### 4.1 Data acquisition and preprocessing

We apply dyna-LOCUS to analyze the resting-state fMRI (rs-fMRI) data from the Philadelphia Neurodevelopmental Cohort (PNC) project (Satterthwaite et al., 2014,^2016^). Children and adolescents between 8 and 21 years at enrollment underwent multimodal neuroimaging which included rs-fMRI. Images were acquired using a single 3T Siemens TIM Trio whole-body scanner. Resting-state fMRI scans were acquired on a single-shot, interleaved multi-slice, echo planar imaging (GE-EPI) sequence. The nominal voxel size is3×3×3mm with full brain coverage achieved with parameters of TR/TE = 3000/32 ms, flip = 90°, and FOV = 200 x 220 mm. Detailed descriptions of the inclusion and exclusion criteria, and the settings for the scanning session can be found inSatterthwaite et al. (2014).

Prior to analysis, we perform quality control procedures on the rs-fMRI. We remove subjects who had more than 20 volumes with relative displacement>0.25mmto avoid images with excessive motion (Satterthwaite et al., 2015;Wang et al., 2016). In total, 514 participants’ rs-fMRI data meet the quality criterion and are used in our analysis. Among these subjects, 289 (56%) are female and the mean age is 15.28 years (SD = 3.11). For rs-fMRI preprocessing, skull stripping is performed on the T1 images to remove extracranial material. The first four volumes of the functional time series are removed to stabilize the signal, leaving 120 volumes for subsequent preprocessing. The anatomical image is registered to the 8th volume of the functional image and subsequently spatially normalized to the MNI standard brain space. The normalization parameters from MNI space are used for the functional images, which are smoothed with a 6 mm FWHM Gaussian kernel. Motion corrections are applied on the functional images. A validated confound regression procedure (Satterthwaite et al., 2015) is performed on each subject’s time series data to remove confounding factors including motions, global effects, white matter (WM), and cerebrospinal fluid (CSF) nuisance signals. Furthermore, motion-related spike regressors are included to bind the observed displacement. Lastly, the functional time series data are band-pass filtered to retain frequencies between 0.01 and 0.1 Hz, which is the relevant frequency range for rs-fMRI.

### 4.2 Dynamic connectivity analysis using dyna-LOCUS

In this study, we adopt Power’s 264-node brain parcellation system (Power et al., 2011) for connectivity analysis. Each node in this node system is a 10 mm diameter sphere in the standard MNI space representing a putative functional area, and the collection of nodes provides good coverage of the whole brain. To facilitate interpretation, we assign the nodes to the functional networks of Smith’s major resting-state network system (Smith et al., 2009). Specifically, the nodes are assigned to medial visual network (“Med Vis”), occipital pole visual network (“OP Vis”), lateral visual network (“LAT Vis”), default mode network (“DMN”), cerebellum (“CB”), sensorimotor network (“SM”), auditory network (“Aud”), executive control network (“EC”), and right and left frontoparietal networks (“FPR” and “FPL”). For the nodes whose assignments are uncertain in Smith’s system, we interpret them using Power’s resting-state network labels (Power et al., 2011).

For each subject, dynamic connectivity is assessed with the commonly used sliding window approach. We use a tapered window, created by convolving a rectangle (width = 15TRs = 45 seconds) with a Gaussian kernel (σ= 3TRs) and slide in steps of 1TR, resulting in 106 windows. Our specification of the sliding window length of 45 seconds is based on the findings in the existing literature which recommend selecting a window length within the range of 30–60 seconds for fMRI (Hutchison et al., 2013;Shirer et al., 2012). This range of window length is shown to produce robust dynamic connectivity results by striking a balance between ensuring a sufficient number of time points in a window for reliable connectivity estimation and having an adequate number of windows across time to capture dynamic changes in brain connectivity. Within this recommended range, we select a window length of 45 seconds, similar to the one employed in numerous fMRI studies (Allen et al., 2014;Marusak et al., 2017;Yang et al., 2014). We extract the fMRI time series from each node and obtain264×264dynamic connectivity matrices for each subject by evaluating the pair-wise correlations between the node-specific fMRI series in each sliding window. Fisher’s Z transformation is applied to the correlations to obtain the dynamic connectivity data for decomposition.

**Fig. 4.**
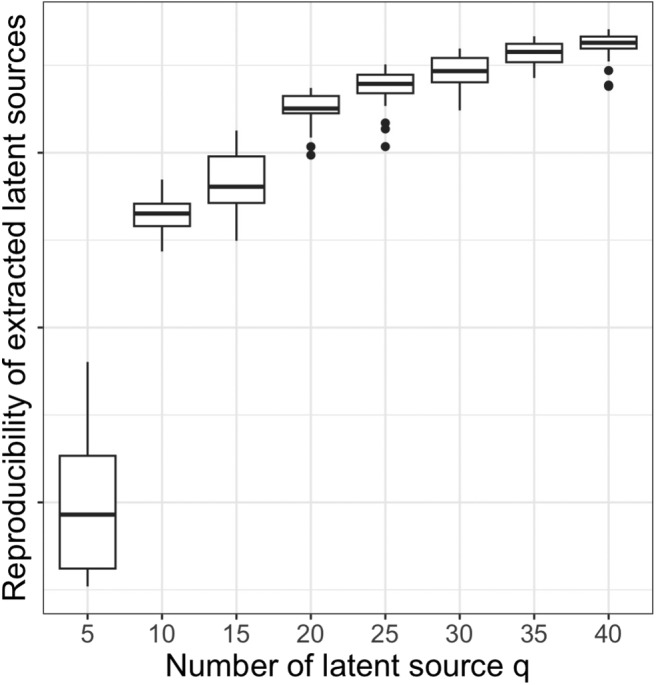
Boxplot of the reproducibility of the extracted latent sources from dyna-LOCUS under different choices of the number of latent sourcesq. The overall reliability across latent sources is close to 0.95 whenqequals 30.

We apply dyna-LOCUS to decompose the dynamic connectivity data to reveal underlying connectivity traits. The choice of the number of latent sourcesqhas been an open topic in blind source separation research. Typically, the number of sources is selected based on a specific objective function or the interpretability of the extracted latent sources. In our study, we selectqbased on the reproducibility and interpretability of the extracted sources. We evaluate the reproducibility of the extracted latent sources for a range ofqvalues (Fig. 4). The reproducibility of the extracted sources initially increases withqand then starts to level off at aroundq= 30. Although the reproducibility still rises slightly withq>30, the interpretability of the extracted sources becomes less ideal whenqbecomes too large. Therefore, we chooseq=30which achieves a good balance between the model size, reproducibility, and interpretability. Using the proposed tuning parameter selection method, we chooseρ=0.95,ϕ=2, andλ=exp(−2)for the dyna-LOCUS optimization.

Regarding the computation time of the analysis, the most computationally intensive phase is the preprocessing step prior to the dyna-LOCUS decomposition, which involves dimension reduction and whitening of the dFC data through singular eigenvalue decomposition (SVD) of dFC across all subjects. The preprocessing of dFC data from the 514 subjects in the PNC study was conducted on the Emory Rollins School of Public Health High-Performance Computing (HPC) cluster, using a single compute node with 32 cores and 256 GB of RAM. This process takes approximately 4 hours to complete. It is worth noting that the preprocessing step is a one-time procedure that only needs to be carried out once for a given dataset. Subsequently, the dyna-LOCUS decomposition of the preprocessed dFC data from the PNC study can be readily executed on a personal laptop. With a specific tuning parameter setting, the dyna-LOCUS decomposition of the PNC data took approximately 1 hour on a MacBook laptop equipped with an Apple M2 Chip and 16 GB of memory. For selecting tuning parameters, one can execute dyna-LOCUS decomposition in parallel for multiple parameter settings, requiring a computation time similar to that of a single decomposition.

### 4.3 Results

#### 4.3.1 Spatial composition of the latent connectivity traits

dyna-LOCUS uncovers 30 dynamic latent connectivity traits as well as the corresponding subject-level temporal trait loadings for each connectivity trait. The mean (SD) of the rank parameter$R_{\ell }$across the latent traits is 4.2 (1.3), with the minimum, median, and maximum values being 2, 4, and 7, respectively. We first present source signal maps that reveal the key brain connections and brain nodes contributing to the connectivity traits. We label the traits in the order of their reproducibility based on the reliability index. Across these 30 traits, the reliability index ranges between 0.31 and 0.94, indicating all traits have at least fair reproducibility. In specific, 3 traits have fair reproducibility, 9 traits have moderate reproducibility, 11 traits have substantial reproducibility, and 7 traits have almost perfect reproducibility. We present the source signal maps for the most reproducible connectivity traits inFigure 5 (Part I and II). The results for all 30 extracted connectivity traits are presented inSection 1of the Supplementary Material. For a comprehensive analysis, we also visualize the 30 dynamic connectivity traits uncovered by connICA and DL in Section 2 of theSupplementary Material. To assess the effect of the window length selection on the results, we conduct a sensitivity analysis by considering alternative window lengths of 30 seconds (10TR) as well as 60 seconds (20TR). We calculate the correlations between the connectivity traits derived from our selected window size of 45 seconds and their matched traits obtained from the alternative window sizes. The median correlation is 0.75 for comparison with the window length of 30 seconds and 0.89 for the window length of 60 seconds, demonstrating a reasonable consistency in findings across varying window lengths.

**Fig. 5.**
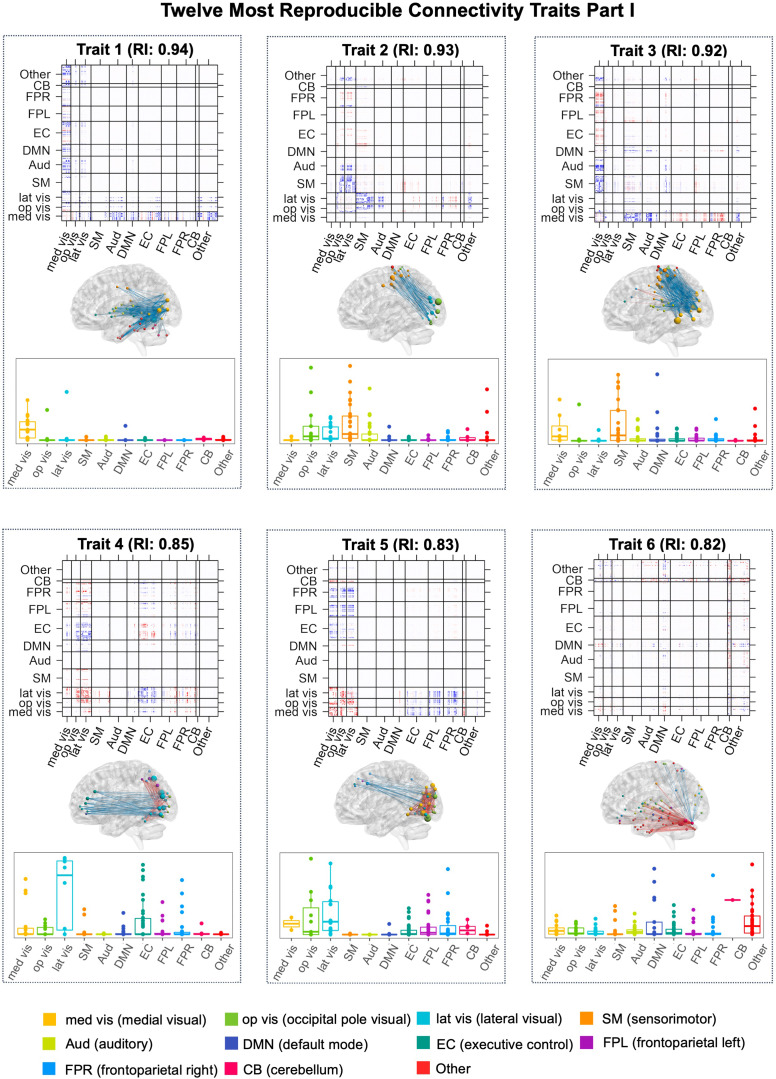

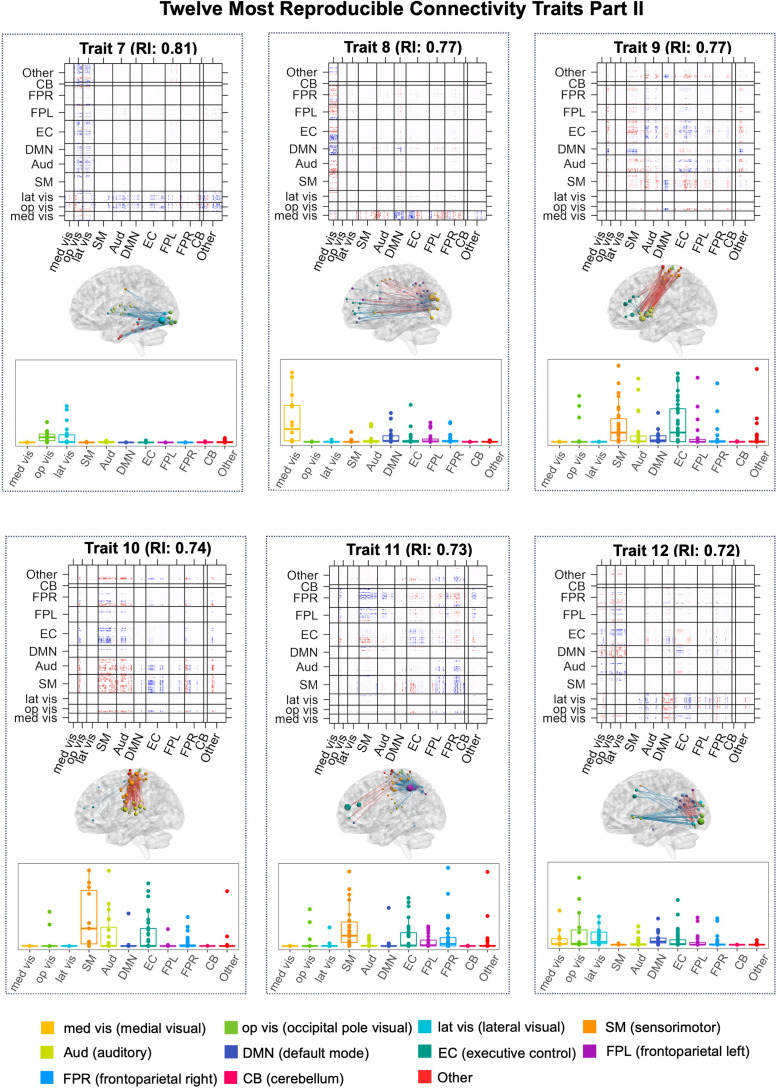
Part I: Twelve most reproducible connectivity traits extracted from the PNC study with reliability index greater than 0.7. The top 0.5% brain connections and significantly expressed nodes based on node contribution index are displayed in the brain maps. Node contribution index that helps identify key brain nodes and networks that drive each connectivity trait are shown in the boxplot arranged by network. Fig. 5. Part II: Twelve most reproducible connectivity traits extracted from the PNC study with reliability index greater than 0.7. The top 0.5% brain connections and significantly expressed nodes based on node contribution index are displayed in the brain maps. Node contribution index that helps identify key brain nodes and networks that drive each connectivity trait are shown in the boxplot arranged by network.

Figure 5presents the 12 most reproducible connectivity traits with a reliability index greater than 0.7. In the figure, the top0.5%brain connections with the highest magnitude of source signal intensity in each of the connectivity traits are mapped onto the brain. Node contribution indices that help identify key brain nodes and networks that drive each connectivity trait are also shown in boxplots arranged by networks. An interesting discovery is that 8 out of the 12 most reproducible traits involve visual networks. This finding aligns well with earlier research (Kang et al., 2011;Zuo et al., 2010), which showed connections involving visual networks are highly reproducible and consistently observed over time across individuals. Results from dyna-LOCUS provide new insights and comprehensive information about the specific neural circuits involving the visual networks that exhibit a remarkably high level of reproducibility in brain connectome, shedding new light on their functional significance and potential implications. In specific, Trait 1 (Med vis-Aud-DMN-EC-CB), Trait 3 (Med vis-SM-Aud-FPR), and Trait 8 (Med vis-DMN-EC-FPL-FPR) are connectivity traits driven by the medial visual (Med vis) network including connections between Med Vis and other brain regions. Trait 2 (Vis-SM-Aud), Trait 4 (Vis-EC), and Trait 7 (Vis-Aud-DMN-EC) mainly feature connectivity between the other two visual networks, i.e. Op Vis/Lat Vis, and other brain regions. Specifically, Trait 2 (Vis-SM-Aud) features connectivity between Op Vis/Lat Vis and the sensory-motor and auditory networks. Trait 4 (Vis-EC) mainly consists of connections within and between the Op Vis and Lat Vis, connections within the executive control network, and connections between Op Vis/Lat Vis and the executive control networks. Trait 7 (Vis-Aud-DMN-EC) mainly consists of connections between nodes in Op Vis/Lat Vis and nodes in auditory, DMN, and executive control networks. Trait 5 (Vis-FPR-FPL) mainly consists of connections within the three visual networks and connections between the visual networks and other brain regions, i.e. executive control, left and right frontoparietal, and cerebellum networks. Trait 12 (Vis-DMN-EC) involves connectivity between the three visual networks and cognitive networks, particularly DMN and executive control networks.

Among the 12 most reproducible traits, Trait 6 (CB) is a cerebellum-driven connectivity trait including connections within the cerebellum and also between the cerebellum and many other brain networks such as auditory, visual, DMN, executive control, etc. Cerebellum is traditionally known for its role in movement coordination and motor learning, but recently increasing evidence suggests it may also be involved in higher-order functions, including emotional and cognitive processing (Beuriat et al., 2020;Schmahmann, 2004). Our finding provides exciting new evidence that the cerebellum has highly consistent neural connections with not only motor and sensory networks but also with higher-order emotional and cognitive networks in brain dynamic connectome. In addition, the source signal maps of dyna-LOCUS allow investigators to identify specific brain regions that have highly reliable connections with the cerebellum, which provide valuable information to advance the new understanding of this important part of the brain. For the rest of the 12 most reproducible traits, Trait 9 (SM-Aud-DMN-EC) involves connections within and between sensory-motor, auditory, DMN, and executive control networks. Trait 10 (SM-Aud-EC) is driven by the sensory-motor and auditory networks, and their connections with the executive control network. Trait 11 (FPR-FPL-SM-EC) mainly consists of the connections within the frontal partial networks and their connection with other brain networks.

#### 4.3.2 Temporal expression of the latent connectivity traits

**Fig. 6.**
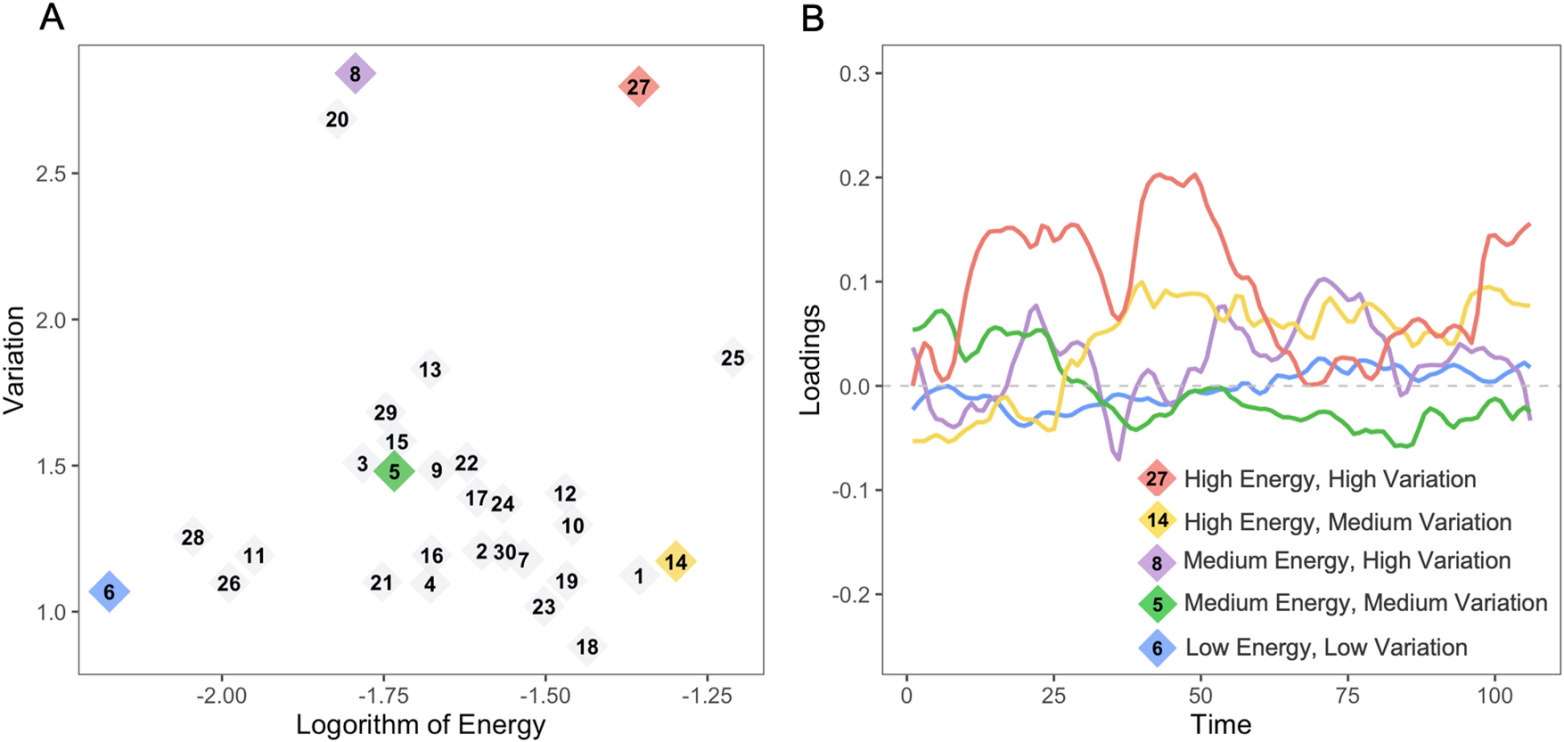
(A) Averaged variation against log(energy) of connectivity traits across subjects. Colored traits represent several types of energy and variation patterns including high-energy and high-variation (red), high-energy and medium-variation (yellow), medium-energy and high-variation (purple), medium-energy and medium-variation (green), and low-energy and low-variation (blue). (B) Temporal loading series of representative traits from sample subjects: high-energy and high-variation (Trait 27), high-energy and medium-variation signal (Trait 14), medium-energy and high-variation (Trait 8), medium-energy and medium-variation (Trait 5), and low-energy and low-variation (Trait 6).

In addition to unveiling the spatial composition of connectivity traits, dyna-LOCUS also generates trait loadings that capture the temporal expression of these traits in brain dynamic connectome. These trait loadings offer new insights into the distinctive features exhibited by each connectivity trait throughout its dynamic profiles (Fig. 6).Figure 6Aplots the variation against the logarithm of energy of each connectivity trait. Our analysis reveals the presence of certain traits that exhibit distinctive characteristics in terms of one or both of these measures. We observe a diverse range of energy and variation patterns across these identified traits, encompassing distinct combinations of high-energy and high-variation, high-energy and medium-variation, medium-energy and high-variation, medium-energy and medium-variation, as well as low-energy and low-variation. These traits are denoted with different colors inFigure 6A. InFigure 6B, we display the trait loading series of these traits, and their source signal maps are presented inFigure 7. Among these traits, Trait 27 (SM-DMN-Aud) demonstrates particular high-energy and high-temporal variation. It mainly consists of connections between the sensorimotor network and other regions, especially the default mode network. Its temporal loading series inFigure 6Breveals that it is highly expressed during certain periods and then not expressed in others. On the other hand, Trait 6 (CB), the cerebellum-driven trait, demonstrates the lowest energy and also low variability across the scanning session, indicating it is a stable trait with a modest expression level across time. As another type of pattern, Trait 14 (EC-Aud-DMN-FPR), which includes connections within executive control and also between executive control and auditory, default mode, and frontal-parietal right networks, demonstrates high energy and only medium variation. The temporal loading series inFigure 6Breveals Trait 14 remains at a high level of expression for a good proportion of time during the scanning and is more stable in its temporal expression as compared with other high-energy connectivity traits, indicating it is generally highly expressed across time. Trait 5 (Vis-FPR-FPL), which involves the frontal-parietal networks and their connections with other networks, exhibits medium energy and medium variation. It displays intermittent expression across scans, but not very frequently switching between the on and off status. Finally, Trait 8 (Med vis-DMN-EC-FPL-FPR), driven by the medial visual network, has medium-energy and high-variation. The loading pattern for this trait shows frequent switches between no expression and exhibiting expression.

**Fig. 7.**
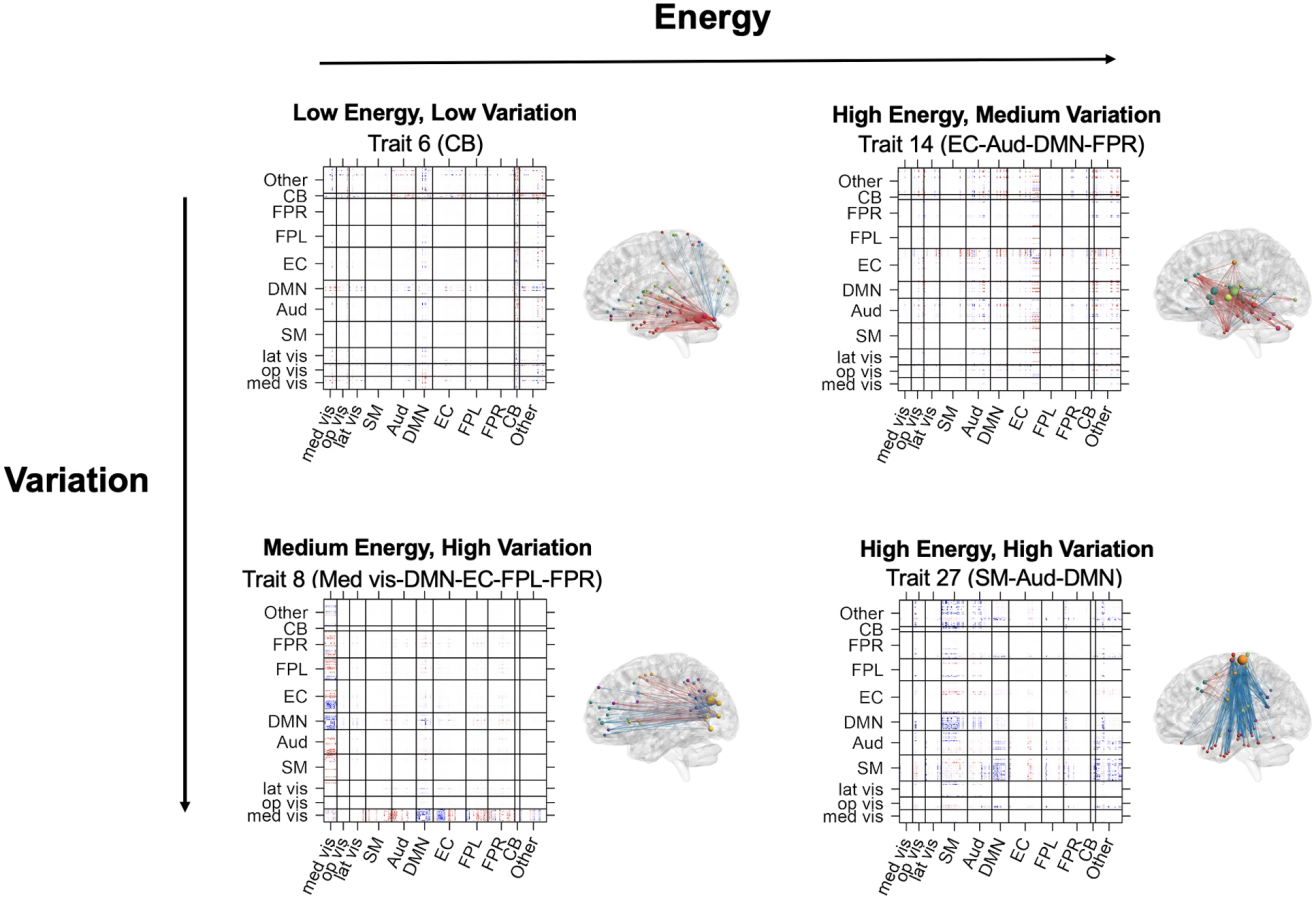
Connectivity traits with different types of energy and variation patterns. The top 0.5% brain connections and significantly expressed nodes based on the node contribution index are depicted in the brain maps.

**Fig. 8.**
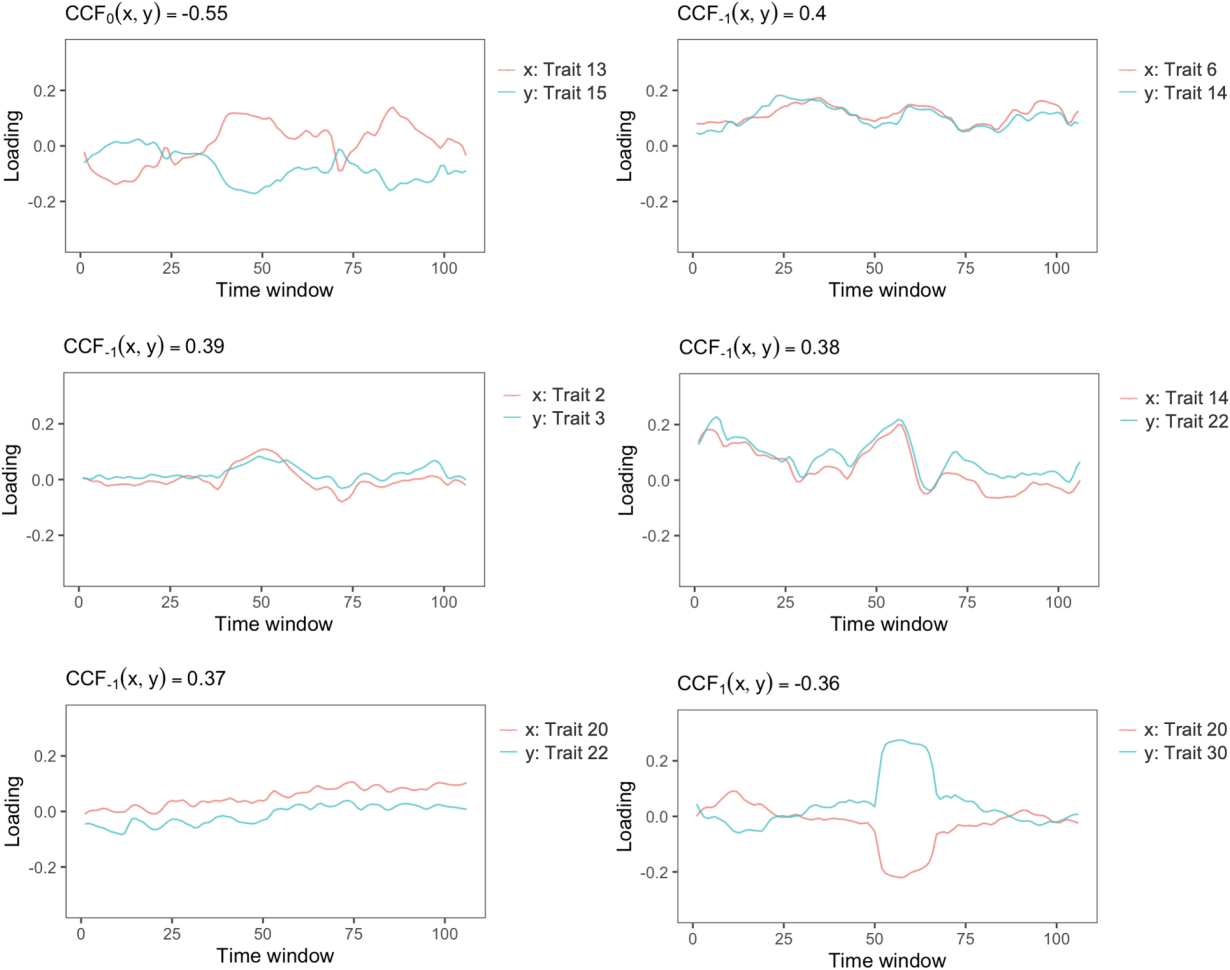
Temporal loading time series of six synchronized connectivity trait pairs from example individuals. Median CCF across individuals is displayed for each trait pair. Traits with higher reproducibility in each pair is shown in red and traits with lower reproducibility in a pair is in blue.

In addition to examining the temporal expression levels of each trait, we also investigate the interaction and synchronization between connectivity traits using the CCF measure. Every pair of connectivity traits exhibits their peak synchronization at a lag of either one or zero, indicating the rapid interactions and synchronization between these traits. This finding suggests that the expression levels of these connectivity traits are characterized by swift and efficient information exchange, facilitating seamless coordination and integration within the brain connectome. Among all pairs of connectivity traits, we identify six pairs that demonstrate a strong relationship with a median CCF magnitude greater than 0.35 across individuals.Figure 8displays trait loading series of the trait pairs of example individuals to illustrate their synchronization. For example, Trait 13 (EC-FPL) and Trait 15 (Vis-SM-Aud) have a median CCF of -0.55 with a lag of zero, indicating that these two traits tend to be synchronized in the opposite direction, which is observed in their trait loading series inFigure 8. Trait 6 (CB) and Trait 14 (EC-Aud-DMN-FPR) have a median CCF of 0.40 with a lag of -1, indicating they are synchronized in the same direction and Trait 14 is leading.

#### 4.3.3 Gender and age differences in the connectivity traits

dyna-LOCUS offers an efficient and convenient method for modeling brain–behavior relationships, enabling investigation of variations in connectivity traits linked to demographics, behavior, and disease. This is achieved through analyzing associations between individual trait loadings obtained from dyna-LOCUS and an individual’s demographic attributes, behavior, or clinical symptoms.

**Fig. 9.**
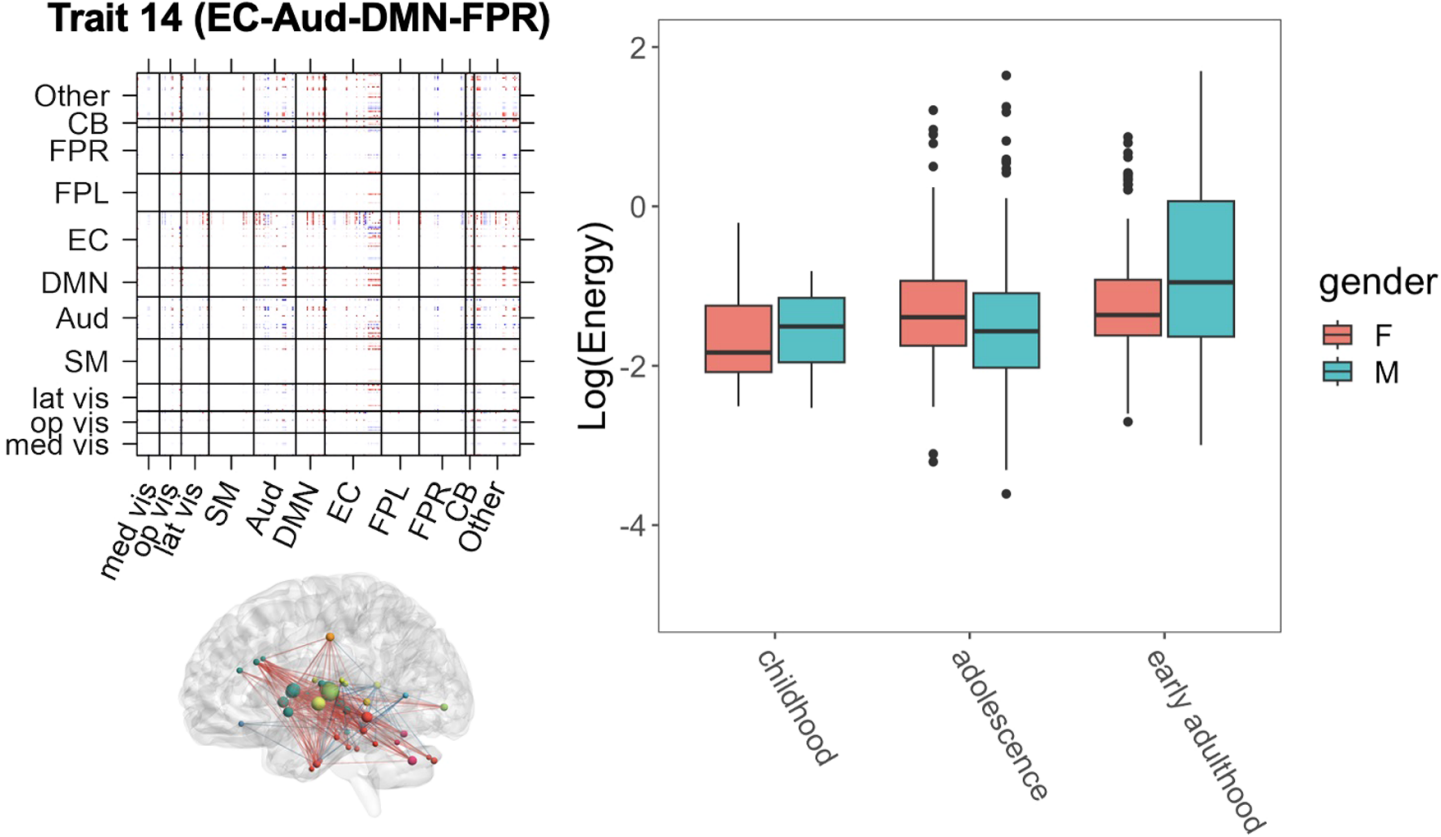
Gender differences in the development of an executive function-related connectivity Trait 14 (EC-Aud-DMN-FPR). The left of the figure displays the connectivity source signals and its brain map (showing the top0.5%brain connections). The right of the figure displays the logarithmic energy of the trait’s temporal expression for males and females across three age groups: middle and late childhood (ages 8–11 years), adolescence (ages 12–17 years), and early adulthood (ages 18–21 years). Males and females demonstrate different developmental patterns for this trait.

In the PNC study, we are interested in exploring the maturation of neural circuits throughout adolescence and potential developmental differences between genders. Previous neurodevelopmental studies have reported developmental changes in the executive function and network from childhood to adolescence (Best & Miller, 2010;Chai et al., 2017). Here, we leverage results from dyna-LOCUS analysis of the PNC study to investigate age and gender differences in the connectivity traits involving executive function. To this end, we categorize participants into three age groups: middle and late childhood (ages 8-11), adolescence (ages 12-17), and early adulthood (ages 18-21), with 69, 291, and 143 participants in each group, respectively. We then employ regression models to investigate the relationship between the logarithm of energy of individual’s trait loadings on Trait 14 (EC-Aud-DMN-FPR) (Fig. 9), which is primarily driven by the executive function network, and the individual’s gender and age group.

Trait 14 (EC-Aud-DMN-FPR) consists of connections within the executive control (EC) network and connections between the executive control network and the auditory, default mode, and frontal-parietal right networks. Our analysis of the traits’ temporal expression insection 4.3.2. shows that Trait 14 (EC-Aud-DMN-FPR) is a stable and high-energy connectivity trait that is often highly expressed across time (Fig. 6), indicating the significance of this executive function-related connectivity trait in the brain dynamic connectome. The association analysis between its trait loadings and individuals’ age and gender reveals the energy, representing the expression level of the trait, increases with age in both gender groups (Fig. 9). This observation suggests that the presence and prominence of this particular trait become more pronounced during the process of neurodevelopment in childhood and adolescence. An interesting finding from our analysis is that males and females demonstrate distinctive developmental trajectories for this trait, with a significant gender×age interaction (p=0.03). Specifically, females exhibit a notable increase in energy from late childhood to adolescence, and then a gradual upswing from adolescence to early adulthood. In contrast, males show relatively stable energy levels for Trait 14 between late childhood and adolescence, followed by a substantial increase upon entering early adulthood. This indicates the expression level of Trait 14 reaches higher levels earlier in females compared with males. To assess the reliability of this finding, we implement the data resampling validation method to evaluate the reproducibility of the result using replication samples generated through data resampling. The results provide supporting evidence for the robustness of the significant interaction effect between age and gender in the neurodevelopment of Trait 14 (Section 3 of theSupplementary Material). Notably, as young adults, males display significantly higher expression levels of Trait 14 compared with females (p<0.01), indicating that Trait 14 is more prominent among young adult males. The gender difference in the expression level of Trait 14 is not significant during childhood (p=0.782) and adolescence (p=0.113).

#### 4.3.4 Whole-brain dFC states

We implement the new dyna-LOCUS pipeline to identify whole-brain dFC states in the PNC study. After obtaining results from dyna-LOCUS, we perform clustering analysis on the trait loadings to identify brain state cluster centroids. To properly initialize the clustering analysis, we adopt an initializing strategy similar to the one in the previous dFC state analysis (Allen et al., 2014). Specifically, we randomly select 20 trait loading vector exemplars from each subject and perform clustering on the set of subject exemplars. This procedure is repeated 100 times with random initializations in order to escape local minima. The resulting centroids based on subject exemplars are then used to initialize the clustering of all trait loading data. We determine the number of clusters by applying the elbow criterion to the ratio between the within-cluster distance and the between-cluster distance (Thorndike, 1953). After obtaining the trait loading centroids based on all data, we reconstruct whole-brain dFC states by multiplying the centroid trait loadings with the connectivity trait basis matrices derived from dyna-LOCUS. The top panel ofFigure 10displays the whole-brain dFC states, their percentage of occurrence, and their corresponding trait loading centroids for the PNC study. As a comparison, we derive the dFC states by directly clustering observed dFC matrices using the procedure inAllen et al. (2014). Following their paper, we randomly select six dFC matrices exemplars from each subject and perform clustering on all subjects’ exemplars. This procedure is repeated 100 times with random initializations to escape local minima. The resulting centroids are utilized to initialize the clustering of all dFC matrices. The number of clusters is chosen using the approach inAllen et al. (2014). The bottom panel ofFigure 10illustrates the clustering results using Allen’s method.

**Fig. 10.**
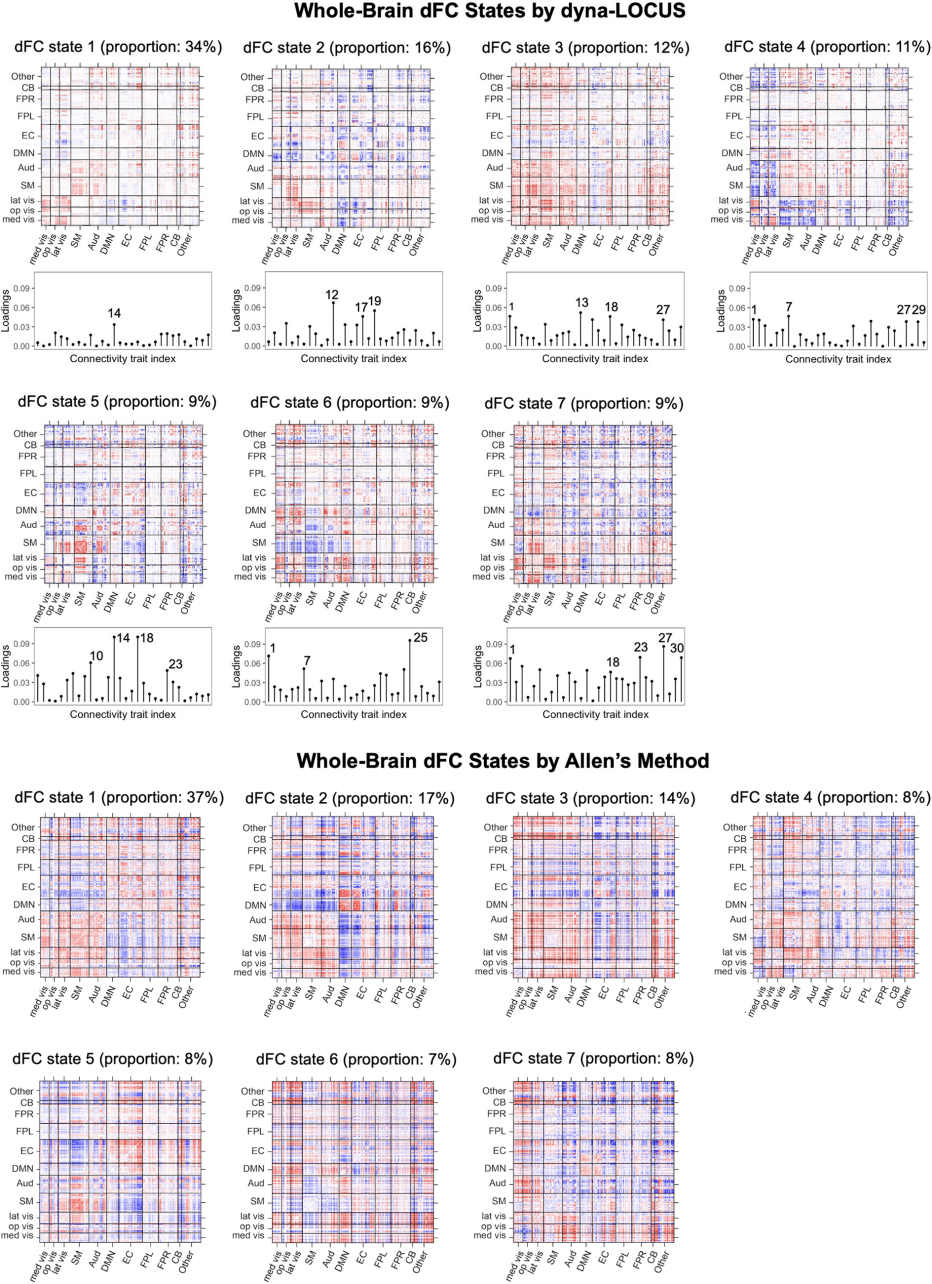
Whole-brain dynamic functional connectivity (dFC) states estimated by dyna-LOCUS and Allen’s method, along with the corresponding proportions of their occurrence. dyna-LOCUS also provides the loadings of the underlying connectivity traits in each of the dFC states.

Our findings demonstrate a notable level of concordance between the results obtained by dyna-LOCUS and Allen’s method. Specifically, both methods identify seven whole-brain dFC states. We are able to readily match each of the dFC states identified by dyna-LOCUS with a corresponding dFC state identified by Allen’s method, based on their connectivity patterns. Additionally, we observed a high degree of consistency in the percentage of occurrence of the matching dFC states between the two methods. The correspondence between the results obtained by dyna-LOCUS and Allen’s method, which is a well-established approach for identifying dFC states, provides validation that the novel dyna-LOCUS pipeline is a reliable method for identifying whole-brain dFC states.

Our new dyna-LOCUS pipeline offers several key advantages over existing methods. Firstly, by clustering the low-dimensional trait loadings obtained from dyna-LOCUS, our method achieves a substantial improvement in computational efficiency compared with existing methods that perform clustering on the original dFC matrices. For example, for the PNC study, Allen’s method takes 8 hours and 26 minutes to conduct clustering analysis on the264×264dFC matrices, whereas our dyna-LOCUS pipeline uses only 15.73 seconds to perform the analysis on the30×1trait loading vectors. Secondly, benefiting from dyna-LOCUS’s sparsity regularization, our pipeline generates much sparser dFC states as compared with the existing method. Therefore, the new pipeline is able to reveal the most relevant edges in the dFC states, while effectively filtering out a large amount of less crucial connections. This results in more parsimonious and interpretable representations of the dFC states. Thirdly, our pipeline goes beyond solely identifying the dFC states; it also provides valuable information on the corresponding trait loadings. These trait loadings elucidate the contribution of the underlying connectivity traits to the whole-brain dFC states, revealing the key connectivity traits that drive each specific dFC state. Hence, researchers can gain deeper insights into the specific neural mechanisms and processes associated with different dFC states.

#### 4.3.5 Comparison between the static and dynamic FC analysis

In this section, we compare the results between the static FC analysis using LOCUS (Wang & Guo, 2023) and the dynamic FC analysis using the proposed dyna-LOCUS. LOCUS assumes brain functional connectivity (FC) is stationary and extracts latent sources underlying static FC measures obtained using the whole fMRI BOLD series (Wang & Guo, 2023), ignoring changes in brain connectivity over time. In comparison, dyna-LOCUS models dynamic changes in functional connectivity and uncovers latent sources underlying the series of dynamic FC measures obtained using fMRI BOLD signals within short time windows that slide across the session.

**Fig. 11.**
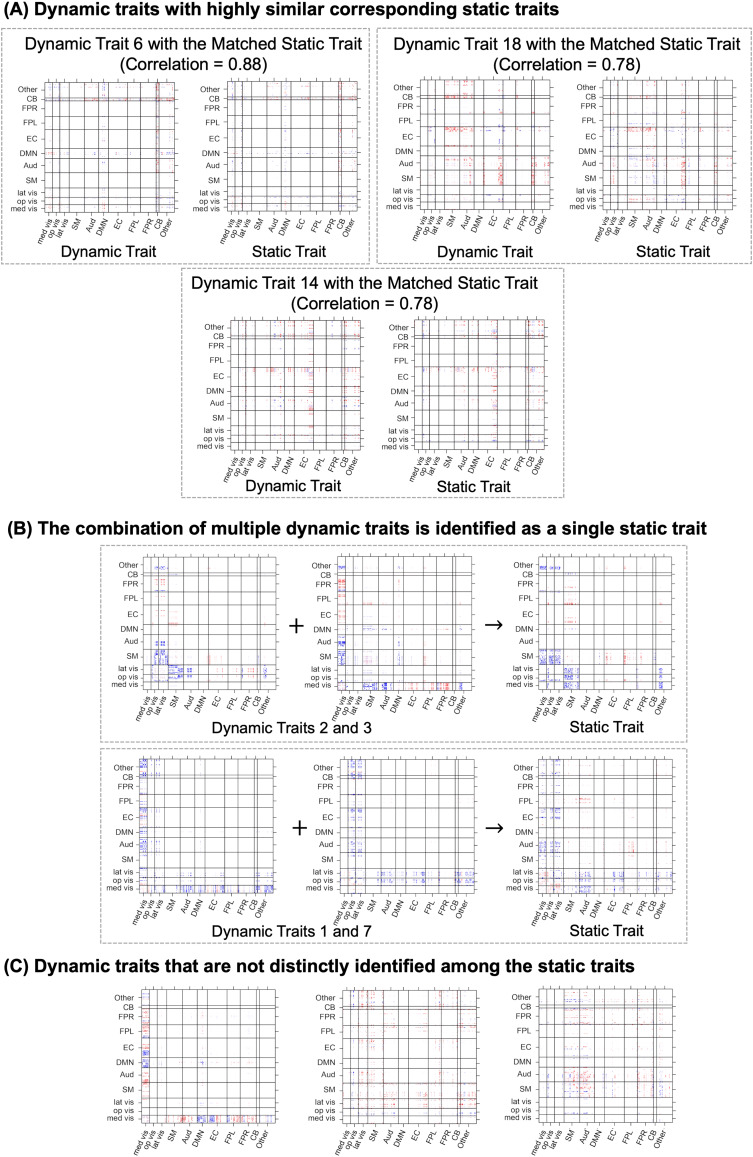
(A) Three dynamic traits (Traits 6, 14, and 18) with highly similar static trait matches. (B) Examples of static traits representing combinations of multiple dynamic traits. (C) Examples of dynamic traits that are not clearly identified among static traits.

We compare the latent connectivity sources extracted from the LOCUS and dyna-LOCUS from the PNC study. The static connectivity traits extracted from the PNC study using LOCUS are presented in Section 6 of theSupplementary Material. Our comparison reveals both similarities and differences in the results from the two methods. For certain dynamic latent sources extracted by dyna-LOCUS, we identify corresponding static latent sources extracted from LOCUS, which exhibit similar spatial compositions.Figure 11Aillustrates three dynamic traits from dyna-LOCUS (Traits 6, 14, and 18) that have the most similar corresponding static traits from LOCUS. The temporal trait loading analysis of dyna-LOCUS (Fig. 6) reveals that these dynamic traits display a low or medium level of variation in their temporal expression. This suggests a consistently stable presence over time in the brain connectome, elucidating why these dynamic traits closely resemble static traits identified by LOCUS. Furthermore, we notice situations where a combination of multiple dynamic traits is identified as a single static trait. For instance, the combination of dynamic Trait 2 and Trait 3 corresponds to a static trait identified by LOCUS (Fig. 11B). The temporal trait loading analysis (Fig. 8) reveals that Traits 2 and 3 exhibit strong synchronization in their temporal expression with a medium CCF of 0.39 across subjects. This synchronization explains their aggregation as a single static trait in the LOCUS analysis. This aggregation phenomenon also occurs for dynamic Traits 1 and 7 (Fig. 11B). In addition to these consistent findings, dyna-LOCUS also unveils dynamic traits that are not distinctly identified among the static traits extracted by LOCUS (such as dynamic Traits 8, 15, and 29 displayed inFigure 11C). The temporal trait loading analysis of dyna-LOCUS (Fig. 6) reveals that these dynamic traits exhibit high variation in their temporal expression. This variability suggests that their presence across time fluctuates significantly, potentially accounting for why these dynamic traits are not distinctly identified as static traits by LOCUS. The similarities and differences observed in the results from dyna-LOCUS and LOCUS highlight the potential of dyna-LOCUS to offer novel insights into brain functional connectivity beyond the static FC findings obtained by LOCUS.

## 5 Discussion

In this paper, we propose a general latent source separation method for brain dynamic connectome. There has been a strong interest in advancing the understanding of the dynamic reorganization of the human brain. The proposed dyna-LOCUS method aligns with the broader framework of analyzing dFC using clustering methods such as k-means or decomposition methods such as ICA and PCA to unveil basis patterns underlying the observed dynamic connectome (Miller et al., 2016). However, the distinguishing features of dyna-LOCUS set it apart from the existing methods, enabling dyna-LOCUS to offer valuable contributions to the field. Specifically, compared with the clustering methods such as k-means or fuzzy k-means, dyna-LOCUS is tailored toward a distinct objective and produces different types of results. k-means methods cluster the observed dFC data into a number of clusters, where the cluster centroids represent specific “states” of the overall whole-brain connectivity patterns observed over time. In comparison, dyna-LOCUS decomposes the observed dFCs to reveal the latent connectivity sources/traits underlying the overall whole-brain connectivity, where each trait represents a subset of brain connections that tend to occur together during neural processing. Furthermore, dyna-LOCUS uncovers the temporal expression profiles of the latent connectivity traits. Therefore, results from dyna-LOCUS provide new information to reveal the underlying connectivity traits that constitute the whole-brain connectivity “states” and further reveal the key connectivity traits that drive each whole-brain connectivity “state.” Compared with decomposition methods such as ICA or PCA, dyna-LOCUS shares a similar goal of decomposing dFC matrices to reveal underlying latent sources. However, dyna-LOCUS incorporates several innovative methodology strategies that lead to advancements over the existing ICA/PCA methods. Specifically, dyna-LOCUS incorporates innovations such as a low-rank structure to improve estimation efficiency and accuracy, an angle-based sparsity regularization for reliably recovering source signal maps by reducing spurious edges, and a temporal smoothness regularization to improve the estimation of temporal expression profiles of connectivity traits. A related work that models dFC using sparse low-rank matrices isCai et al. (2017). In this work,Cai et al. (2017)decompose dFC as a linear combination of a set of dynamic sparse connectivity patterns (dSCPs), modeled via rank-one matrices with sparsity imposed on the rank-one vector, the dSCPs. There are several distinctions between dyna-LOCUS and dSCPs. In comparison with dSCPs that use rank-one matrices, dyna-LOCUS uses a more flexible low-rank factorization where the rank parameters are source specific to accommodate different types of connectivity traits and can be selected using our proposed adaptive selection method. To achieve sparsity, dyna-LOCUS utilizes a novel angle-based element-wise sparsity regularization, specifically designed to achieve sparsity in the connectivity sources. Furthermore,Cai et al. (2017)focus on identifying whole-brain dFC states which are combinations of the underlying connectivity sources and investigating group differences in these dFC states. Hence,Cai et al. (2017)select the number of sources based on classification accuracy for subject groups. In comparison, our paper focuses on reliably uncovering latent connectivity sources and revealing their temporal dynamics, interactions, and synchronization with our proposed temporal metrics. We select the number of sources based on the reproducibility of the extracted sources.

Another related method is the recently proposed LOCUS method for decomposing static functional connectivity matrices (Wang & Guo, 2023). Both dyna-LOCUS and LOCUS are designed to investigate brain functional connectivity (FC) and share similarities such as modeling connectivity patterns using a low-rank structure and sparsity regularization. However, the two methods have major differences in terms of the type of functional connectivity data the methods are applied to, the results generated from the methods, and the analytical approaches. LOCUS is designed for decomposing stationary functional connectivity measures that are derived based on the whole fMRI time series in a scanning session, ignoring the temporal changes in brain connectivity. In comparison, dyna-LOCUS uncovers latent sources underlying the series of dynamic FC measures obtained using fMRI BOLD signals within short time windows that slide across the session. In addition to recovering the spatial compositions of the underlying dynamic connectivity sources/traits, dyna-LOCUS also produces temporal trait loading series and several novel temporal dynamic metrics to measure the energy and variation of each dynamic trait and to reveal the interaction and synchronization between traits. Furthermore, dyna-LOCUS offers an efficient and reliable approach to identifying whole-brain dynamic connectivity states and elucidating the contribution of each connectivity trait to the dynamic FC states. These temporal dynamic insights into functional connectivity are not attainable using the LOCUS method. There are also technical differences between the two methods. dyna-LOCUS models the temporal expression profile of each connectivity trait and incorporates temporal smoothness regularization. This leads to variations in optimization strategies, particularly for updating the loading matrix in the decomposition model. The LOCUS method updates the trait loading matrix using a simple regression method by regressing subjects’ static FC against the estimated static sources. In comparison, dyna-LOCUS updates its temporal loading matrix using a new and more sophisticated strategy that takes into account the temporal smoothness regularization (Appendix B). The aforementioned distinguishing features underscore the unique capabilities of dyna-LOCUS in probing dynamic connectivity, thereby offering novel contributions beyond the LOCUS method.

Our dyna-LOCUS analysis of rs-fMRI data from the PNC study has led to exciting insights into the latent sources that underlie the brain’s dynamic functional connectome. Among the 30 dyna-LOCUS-extracted latent connectivity traits, an impressive 18 of them demonstrate substantial or almost perfect reproducibility with the resampling of study participants. These traits represent consistent subsystems in neural processing and brain organizations, reflecting the cohesive interactions among specific neural circuits. The interplay among these subsystems results in the dynamic reconfiguration of the brain’s functional connectivity patterns over time. The sparse source signal maps generated by dyna-LOCUS allow us to reliably identify the key neural connections and brain nodes that drive each of these subsystems. In addition to unveiling the composition of the connectivity traits, dyna-LOCUS offers important insights into the temporal expression characteristics of each trait, providing a deeper understanding of how these traits manifest over time. For instance, Trait 6 (CB), known for its cerebellum-driven nature, emerges as a stable trait with a modest expression level across time. This stability suggests a consistent and enduring involvement of the cerebellum-driven connectivity pattern in the brain’s dynamic organization. In contrast, Trait 27 (SM-DMN-Aud), encompassing long-distance connections between the sensorimotor and DMN networks, exhibits both the highest energy and the greatest temporal variation. This trait displays periods of heightened expression, followed by periods where it becomes less prominent or even disappears. This dynamic behavior suggests the presence of transient interactions between the sensorimotor and DMN networks, which may play crucial roles during specific cognitive processes or behavioral states. The identification of such distinct dynamic features within connectivity traits sheds light on the complexity and diversity of brain dynamics. Understanding the temporal expression patterns of connectivity traits enriches our knowledge of how different brain networks interact and adapt over time, unveiling the intricate mechanisms that underlie various cognitive functions and behaviors.

The dyna-LOCUS analysis of the PNC study has uncovered previously unknown latent connectivity sources within the dynamic connectome. Particularly noteworthy is the discovery of Trait 14 (EC-AUD-DMN-FRP), which is mainly driven by the executive control network and its connections with other cognitive networks. This executive connectivity subsystem exhibits the highest expression level among the connectivity traits, and it also demonstrates the best stability among the highly expressed traits. These findings highlight the consistent and prevalent presence of this executive connectivity subsystem throughout different time points in the dynamic functional connectome. Additionally, the executive connectivity subsystem demonstrates significant interaction with other connectivity traits. Notably, Trait 14 is involved in two out of the top six synchronized pairs of connectivity traits (Fig. 8), indicating its active cooperation with other connectivity subsystems within the connectome. Moreover, our analysis has uncovered seven distinct whole-brain dFC states (Fig. 10). Among these states, dFC state 1 emerges as the most frequently observed, with Trait 14 standing out as the most influential connectivity subsystem, displaying the highest expression level among all connectivity traits in this dFC state. These findings collectively emphasize the central role of Trait 14, the executive connectivity subsystem, in the dynamic functional connectome. Upon conducting a deeper investigation into the development of Trait 14, we made an intriguing discovery regarding distinct developmental trajectories between genders. In females, early development of this executive connectivity subsystem is observed, whereas in males, its development occurs later, mostly from adolescence to early adulthood. As young adults, males display a more pronounced presence of the executive connectivity subsystem compared with females. This gender-specific variation adds another dimension to our understanding of the executive connectivity subsystem.

One potential limitation of the proposed method is that it decomposes the dFC measures as a linear combination of latent connectivity traits, following the common assumption in blind source separation. However, this assumption may not always be able to capture the complexity of real-world systems. A valuable direction for future work is to extend dyna-LOCUS to accommodate nonlinear mixing cases, thereby broadening its applicability across diverse data scenarios and enhancing its capacity to capture complex relationships. Another potential extension to dyna-LOCUS is to incorporate spatial information of the nodes into modeling the latent coordinates of the nodes in the low-rank factorization. This can help further increase the accuracy and reliability in recovering the connectivity traits by taking into account the spatial dependence between the nodes.

## Supplementary Material

Supplementary Material

## Data Availability

The PNC study data are publicly available to download from the database of Genotypes and Phenotypes (dbGaP) via Authorized Access. To request data access, investigators can login the dbGaP controlled-access portal athttps://www.ncbi.nlm.nih.gov/projects/gap/cgi-bin/study.cgi?study_id=phs000607.v3.p2and submit a Data Access Request. The software package for dyna-LOCUS is available athttps://github.com/Emory-CBIS/dynaLOCUS.

## Appendix A. The Derivation of the Final Optimization Function

The original objective function for dyna-LOCUS model with the sparsity and temporal smoothness regularization is

$$\begin{array}{l} \begin{array}{ccc} \text{min}\sum_{i=1}^{N}||{\text{Y}}_{i}-{\text{A}}_{i}\text{S}{||}_{F}^{2}+\phi \sum_{\ell =1}^{q}\sum_{u<v}\left|{\text{x}}_{\ell }\left(u\right)\prime {\text{D}}_{\ell }{\text{x}}_{\ell }\left(v\right)\right| & & \end{array}\\ \;\;\;\;\;\;\;+\lambda \sum_{i=1}^{N}\sum_{t=2}^{T}||{\text{a}}_{it}-{\text{a}}_{i\left(t-1\right)}|{|}_{2}^{2}, \end{array}$$
(A1)

where$\text{S}=\left[\begin{array}{c} {\text{s}}_{1}^{\prime }\\ \ldots \\ {\text{s}}_{q}^{\prime } \end{array}\right]\in {ℛ}_{q\times p}$and${\text{s}}_{\ell }=ℒ({\text{X}}_{\ell }{\text{D}}_{\ell }{\text{X}}_{\ell }^{\prime })$.

Denote the loading matrix$\text{A}=\{a_{it\ell }\}\in {ℛ}^{NT\times q}$as$\text{A}=[{\text{A}}_{1}^{\prime },...,{\text{A}}_{N}^{\prime }]\prime \in {ℛ}^{NT\times q}$, and each${\text{A}}_{i}=\left[{\text{a}}_{i1},...,{\text{a}}_{iT}\right]\prime$$\in {ℛ}^{T\times q}$, the temporal smoothness regularization term can be written in the matrix form:

$$\sum_{i=1}^{N}\sum_{t=2}^{T}||{\text{a}}_{it}-{\text{a}}_{i\left(t-1\right)}|{|}_{2}^{2}=\sum_{i=1}^{N}||\text{R}{\text{A}}_{i}|{|}_{F}^{2},$$
(A2)

where$\text{R}\in {ℛ}^{(T-1)\times T}$is defined as${\text{R}}_{ij}=1$ifi=j,${\text{R}}_{ij}=-1$ifi=j−1, else${\text{R}}_{ij}=0$. Denote${\text{R}}^{*}=\text{I}⊗\text{R}\in {ℛ}^{N(T-1)\times NT}$, where$\text{I}\in {ℛ}^{N\times N}$, we can further write the temporal smoothness regularization in terms ofA. The original objective function can then be represented as

$$\begin{array}{l} \text{min}{\sum_{i=1}^{N}\sum_{t=1}^{T}\left\|{\text{y}}_{it}-\sum_{\ell =1}^{q}a_{it\ell }ℒ\left({\text{X}}_{\ell }{\text{D}}_{\ell }{\text{X}}_{\ell }^{\prime }\right)\right\|}_{2}^{2}\\ \;\;\;\;\;+\phi \sum_{\ell =1}^{q}\sum_{u<v}\left|{\text{x}}_{\ell }\left(u\right)\prime {\text{D}}_{\ell }{\text{x}}_{\ell }\left(v\right)\right|+\lambda {\left\|{\text{R}}^{*}\text{A}\right\|}_{F}^{2}. \end{array}$$
(A3)

With the preprocessing,$\tilde{\text{Y}}=\text{HY}$with$\text{H}={\left(\Lambda_{\text{q}}-{\tilde{\sigma }}_{q}^{2}\text{I}\right)}^{-1/2}$${\text{U}}_{\text{q}}^{\prime }$. Here,${\text{U}}_{\text{q}},\Lambda_{\text{q}}$contain the firstqeigenvectors and eigenvalues based on singular value decomposition (SVD) ofY. The residual variance,${\tilde{\sigma }}_{q}^{2}$, represents the variability inYthat is not explained by the extractedqlatent sources and is estimated by the average of the smallestNT−qeigenvalues in$\Lambda_{q}$. The preprocessed data$\tilde{\text{Y}}$are of dimensionq×pwhere the columns correspond topconnections in the brain. The whitening in the preprocessing leads to an orthogonal mixing matrix on the reduced space$\tilde{A}$which facilitates the subsequent model estimation. With the preprocessed data and the orthogonal mixing matrix, the final optimization function for dyna-LOCUS is

$$\begin{array}{l} {\text{min}}_{\tilde{A},\left\{X_{\ell },D_{\ell }\right\}}\sum_{l=1}^{q}{\left\|\;\tilde{Y}\prime {\tilde{a}}_{\ell }-ℒ\left(X_{\ell }D_{\ell }X_{\ell }^{\prime }\right)\;\right\|}_{2}^{2}\\ \;\;\;\;\;\;+\phi \sum_{\ell =1}^{q}\sum_{u<v}\left|x_{\ell }\left(u\right)\prime D_{\ell }x_{\ell }\left(v\right)\;\right|+\lambda {\left\|\;W\tilde{A}\;\right\|}_{F}^{2}, \end{array}$$
(A4)

where${\tilde{a}}_{\ell }$is theℓth column of$\tilde{A}$, and$W=R^{*}H^{*}$, with$H^{*}=U_{q}{\left(\Lambda_{q}\;-\;{\tilde{\sigma }}_{q}^{2}I\right)}^{1/2}$being the dewhitening matrix.

## Appendix B. The Estimation Algorithm for dyna -Locus

Based on the block multiconvexity property, we derive an efficient algorithm with closed-form solutions in each updating step. The algorithm estimates the parameters by iterating three updating steps:

**Step 1: Updating** $X_{\ell }$

We propose a node-rotation algorithm that updates$X_{\ell }$at a nodevwhile conditioning on the other nodes and then rotating across the nodes. Since we have${\left\|\;{\tilde{Y}}^{\prime }{\tilde{a}}_{\ell }-ℒ\left(X_{\ell }D_{\ell }X_{\ell }^{\prime }\right)\;\right\|}_{2}^{2}={\left\|\;{\tilde{a}}_{\ell }^{\prime }\tilde{Y}-s_{\ell }^{\prime }\right\|}_{2}^{2}=\sum_{u<v}({\tilde{a}}_{\ell }^{\prime }\tilde{Y}\left(u,v\right)$${-x_{\ell }{\left(u\right)}^{\prime }D_{\ell }x_{\ell }\left(v\right))}^{2}$, we can transform equation (A4) to the edge-wise form.

$$\begin{array}{l} \begin{array}{ccc} {\text{min}}_{\tilde{\text{A}},\left\{{\text{X}}_{\ell },{\text{D}}_{\ell }\right\}}\sum_{\ell =1}^{q}\sum_{u<v}{\left({\tilde{\text{a}}}_{\ell }^{\prime }\tilde{\text{Y}}\left(u,v\right)-{\text{x}}_{\ell }\left(u\right)\prime {\text{D}}_{\ell }{\text{x}}_{\ell }\left(v\right)\right)}^{2} & & \end{array}\\ \;\;\;\;\;\;\;\;+\phi \sum_{\ell =1}^{q}\sum_{u<v}\left|{\text{x}}_{\ell }\left(u\right)\prime {\text{D}}_{\ell }{\text{x}}_{\ell }\left(v\right)\right|+\lambda {\left\|\text{W}\tilde{\text{A}}\;\right\|}_{F}^{2}. \end{array}$$
(B1)

To update${\text{X}}_{\ell }$at thekth iteration, we update the latent coordinate${\text{x}}_{\ell }\left(v\right)$for nodev, conditioning on$\tilde{\text{A}}$,${\text{D}}_{\ell }$, and${\text{X}}_{\ell }\left(-v\right)$estimated from thek−1iteration, where${\text{X}}_{\ell }\left(-v\right)$is the submatrix of${\text{X}}_{\ell }$which comprises the latent coordinates of nodes excludingv. Specifically, we update${\text{x}}_{\ell }\left(v\right)$as follows:

$$\begin{array}{l} \begin{array}{ccc} {\text{min}}_{{\text{x}}_{\ell }\left(v\right)}{\left\|{\tilde{\text{Y}}}_{\left\{v\right\}}^{\prime }{\hat{\tilde{\text{a}}}}_{\ell }-{\hat{\text{X}}}_{\ell }\left(-v\right){\hat{\text{D}}}_{\ell }{\text{x}}_{\ell }\left(v\right)\;\right\|}_{2}^{2} & & \end{array}\\ \;\;\;\;\;\;\;+\phi \sum_{u=1,u\neq v}^{V}|{\hat{\text{x}}}_{\ell }\left(u\right)\prime {\hat{\text{D}}}_{\ell }{\text{x}}_{\ell }\left(v\right)|, \end{array}$$
(B2)

where${\tilde{\text{Y}}}_{\left\{v\right\}}$is aq×(V−1)submatrix of$\tilde{\text{Y}}$which includes the subset of columns in$\tilde{\text{Y}}$that correspond to connections involving nodev.

Denote$b_{\ell \left\{v\right\}}={\hat{X}}_{\ell }\left(-v\right){\hat{D}}_{\ell }x_{\ell }\left(v\right)\in {ℛ}^{V-1}$, objective function (B2) can be rewritten as

$${\text{min}}_{{\text{b}}_{\ell \left\{v\right\}}}{\left\|\;{\tilde{\text{Y}}}_{\left\{v\right\}}^{\prime }{\hat{\tilde{\text{a}}}}_{\ell }-{\text{b}}_{\ell \left\{v\right\}}\right\|}_{2}^{2}\;\;+\;\phi \;\;{\left\|\;{\text{b}}_{\ell \left\{v\right\}}\right\|}_{1}$$
(B3)

We can derive an analytical solution for${\text{b}}_{\ell \left\{v\right\}}$(Fan & Li, 2001) and then obtain the estimate for$x_{\ell }\left(v\right)$as

$$\begin{array}{ccc} {\hat{\text{x}}}_{\ell }^{\left(k\right)}\left(v\right)={\hat{\text{D}}}_{\ell }^{-1}{\left({\hat{\text{X}}}_{\ell }{\left(-v\right)}^{\prime }{\hat{\text{X}}}_{\ell }\left(-v\right)\right)}^{-1}{\hat{\text{X}}}_{\ell }{\left(-v\right)}^{\prime }{\hat{\text{b}}}_{\ell \left\{v\right\}}. & & \end{array}$$

After updating${\text{x}}_{\ell }(v)$, we rotate to the next node and repeat the procedure described above across all nodes to obtain an updated estimate for${\text{X}}_{\ell }$. An advantage of the proposed node-rotation algorithm is that it has analytic solutions and does not need gradient-based numerical approximation which makes it highly efficient and reliable.

**Step 2: Updating** ${\text{D}}_{\ell }$

Our next step is to update the diagonal matrix${\text{D}}_{\ell }$forℓ=1,...,q, given the estimate of${\text{X}}_{\ell }$from thekth iteration and the estimate of$\tilde{A}$from thek−1iteration. Based on the objective function (A4), we update the estimate of the diagonal of${\text{D}}_{\ell }$, i.e.${\text{d}}_{\ell }=\text{diag}\left({\text{D}}_{\ell }\right)$via the following:

$${\text{min}}_{{\text{d}}_{\ell }}{\left\|\;\tilde{\text{Y}}\prime {\hat{\tilde{\text{a}}}}_{\ell }-{\hat{\text{Z}}}_{\ell }{\text{d}}_{\ell }\right\|}_{2}^{2}\;\;+\;\;\phi \;\;{\left\|{\hat{\text{Z}}}_{\ell }\;{\text{d}}_{\ell }\right\|}_{1},$$
(B4)

where${\hat{\text{Z}}}_{\ell }\in {ℛ}^{p\times R_{\ell }}$with therth column of${\hat{\text{Z}}}_{\ell }$being$ℒ\left({\hat{\text{x}}}_{\ell }^{\left(r\right)}{\hat{\text{x}}}_{\ell }^{\left(r\right)\prime }\right)$$\left(r=1,\ldots ,R_{\ell }\right)$. An analytical solution for${\text{d}}_{\ell }$can be derived.

**Step 3: Updating** $\tilde{A}$

In this step, we update the mixing matrix$\tilde{\text{A}}$given the estimates of$\left\{{\text{X}}_{\ell },{\text{D}}_{\ell }\right\}$from thekth iteration. The updated estimate for$\tilde{\text{A}}$is derived as follows:

$${\text{min}}_{\tilde{\text{A}}}f={\left\|\;\tilde{\text{Y}}-\tilde{\text{A}}\hat{\text{S}}\;\right\|}_{F}^{2}+\lambda \;\;{\left\|\;\text{W}\tilde{\text{A}}\;\right\|}_{F}^{2},$$
(B5)

where$\hat{\text{S}}$is based on the estimates of${\text{X}}_{\ell }$and${\text{D}}_{\ell }$from thekth iteration. By setting$\frac{\partial f}{\partial \tilde{\text{A}}}=0$, we have

$$\tilde{\text{A}}\hat{\text{S}}\hat{\text{S}}\prime +\lambda \text{W}\prime \text{W}\tilde{\text{A}}=\tilde{\text{Y}}\hat{\text{S}}\prime .$$
(B6)

We denote the eigen decomposition of$\text{S}{\text{S}}^{\prime }$and${\text{W}}^{\prime }\text{W}$as${\text{Q}}_{1}\Lambda_{1}{\text{Q}}_{1}^{\prime }$and${\text{Q}}_{2}\Lambda_{2}{\text{Q}}_{2}^{\prime },$respectively, where$\Lambda_{1}=\text{diag}\left(\lambda_{1}^{\left(1\right)},...,\lambda_{1}^{\left(q\right)}\right)$and$\Lambda_{2}=\text{diag}\left(\lambda_{2}^{\left(1\right)},\ldots ,\lambda_{2}^{\left(q\right)}\right)$. Equation (B6) can be rewritten as

$${\tilde{\text{A}}}^{*}\Lambda_{1}+\lambda \Lambda_{2}{\tilde{\text{A}}}^{*}={\tilde{\text{Y}}}^{*},$$

where${\tilde{\text{A}}}^{*}={\text{Q}}_{2}^{\prime }\tilde{\text{A}}{\text{Q}}_{1}$, and${\tilde{\text{Y}}}^{*}={\text{Q}}_{2}^{\prime }\tilde{\text{Y}}\hat{\text{S}}\prime {\text{Q}}_{1}$. Thus, the solution for the(i,j)th element in${\tilde{\text{A}}}^{*}$is

$${\tilde{\text{A}}}_{i,j}^{*}=\frac{{\tilde{\text{Y}}}_{i,j}^{*}}{\lambda \lambda_{2}^{\left(i\right)}+\lambda_{1}^{\left(j\right)}}.$$
(B7)

And$\tilde{\text{A}}$is updated as

$${\hat{\tilde{\text{A}}}}^{\left(k\right)}={\text{Q}}_{2}{\tilde{\text{A}}}^{*}{\text{Q}}_{1}^{\prime }.$$
(B8)

The proposed iterative estimation algorithm is summarized inAlgorithm 1.

**Algorithm 1:** An Iterative Node-Rotation Algorithm

**Initial** : Initialize ${\hat{\tilde{\text{A}}}}^{\left(0\right)},\left\{{\hat{\text{X}}}_{\ell }^{\left(0\right)},{\hat{\text{D}}}_{\ell }^{\left(0\right)}\right\}$ based on estimates from existing methods such as connICA.
**repeat**
For ℓ=1...q ,
For v=1,...,V ,
**Step 1. Update** ${\text{x}}_{\ell }\left(v\right)$ :
${\hat{\text{b}}}_{\ell \left\{v\right\}}^{\left(k\right)}=\underset{{\text{b}}_{\ell \left\{v\right\}}\in {ℛ}^{V-1}}{\text{argmin}}{\left\|{\tilde{\text{Y}}}_{\left\{v\right\}}^{\prime }{\hat{\tilde{\text{a}}}}_{\ell }-{\text{b}}_{\ell \left\{v\right\}}\right\|}_{2}^{2}+\phi \;{\left\|{\text{b}}_{\ell \left\{v\right\}}\right\|}_{1}$
${\hat{\text{x}}}_{\ell }^{\left(k\right)}\left(v\right)={\hat{\text{D}}}_{\ell }^{-1}{\left({\hat{\text{X}}}_{\ell }\left(-v\right)\prime {\hat{\text{X}}}_{\ell }\left(-v\right)\right)}^{-1}{\hat{\text{X}}}_{\ell }\left(-v\right)\prime {\hat{\text{b}}}_{\ell \left\{v\right\}}^{\left(k\right)}.$
End for v
**Step 2. Update** ${\text{D}}_{\ell }$ :
${\hat{\text{d}}}_{\ell }^{\left(k\right)}=\text{diag}\left({\hat{\text{D}}}_{\ell }^{\left(k\right)}\right)=\underset{{\text{d}}_{\ell }\in {ℛ}^{R_{\ell }}}{\text{argmin}}{\left\|\tilde{\text{Y}}\prime {\hat{\tilde{\text{a}}}}_{\ell }-{\hat{\text{Z}}}_{\ell }{\text{d}}_{\ell }\right\|}_{2}^{2}+\;\phi {\left\|{\hat{\text{Z}}}_{\ell }{\text{d}}_{\ell }\right\|}_{1},$
End for ℓ
**Step 3. Update** $\tilde{\text{A}}$ : ${\hat{\tilde{\text{A}}}}^{\left(k\right)}={\text{Q}}_{2}^{\left(k\right)}{\hat{\tilde{\text{A}}}}^{*\left(k\right)}{\hat{\text{Q}}}_{1}^{{\left(k\right)}^{\prime }}.$ Perform an orthogonal transformation on ${\hat{\tilde{\text{A}}}}^{\left(k\right)}$ **until** $\frac{∥{\hat{\tilde{\text{A}}}}^{\left(k\right)}-{\hat{\tilde{\text{A}}}}^{\left(k-1\right)}{∥}_{F}}{∥{\hat{\tilde{\text{A}}}}^{\left(k-1\right)}{∥}_{F}}<{\#}_{1}$ and $\frac{∥{\hat{\text{S}}}^{\left(k\right)}-{\hat{\text{S}}}^{\left(k-1\right)}{∥}_{F}}{∥{\hat{\text{S}}}^{\left(k-1\right)}{∥}_{F}}<{\#}_{2}.$
