# Supplementary material for: Unveiling hidden sources of dynamic functional connectome through a novel regularized blind source separation approach
Source: Imaging Neurosci (Camb). 2024 Jul 12;2:imag-2-00220. doi: 10.1162/imag_a_00220 (PMC12272195; doi:10.1162/imag_a_00220)
Supplement: Supplementary Material [file imag_a_00220-supp.pdf]

# SUPPLEMENTARY MATERIALS
## for "Unveiling Hidden Sources of Dynamic Functional Connectome through a Novel Regularized Blind Source Separation Approach"

Jialu Ran, Yikai Wang, and Ying Guo
Department of Biostatistics and Bioinformatics, Emory University

## 1. Latent dynamic connectivity traits extracted from the PNC study using dyna-LOCUS

dyna-LOCUS uncovers 30 dynamic latent connectivity traits by decomposing dynamic functional connectivity (dFC) measures derived from resting state fMRI (rs-fMRI) data from the Philadelphia Neurodevelopmental Cohort (PNC) project. Figure S1 (Part I - Part V) presents the source signal maps for all 30 connectivity traits ranked by their reliability index. In the figure, the top $0.5\%$ brain connections with the highest magnitude of source signal intensity in each of the connectivity traits are mapped onto the brain. Node contribution indices that help identify key brain nodes and networks that drive each connectivity trait are also shown in boxplots arranged by networks.

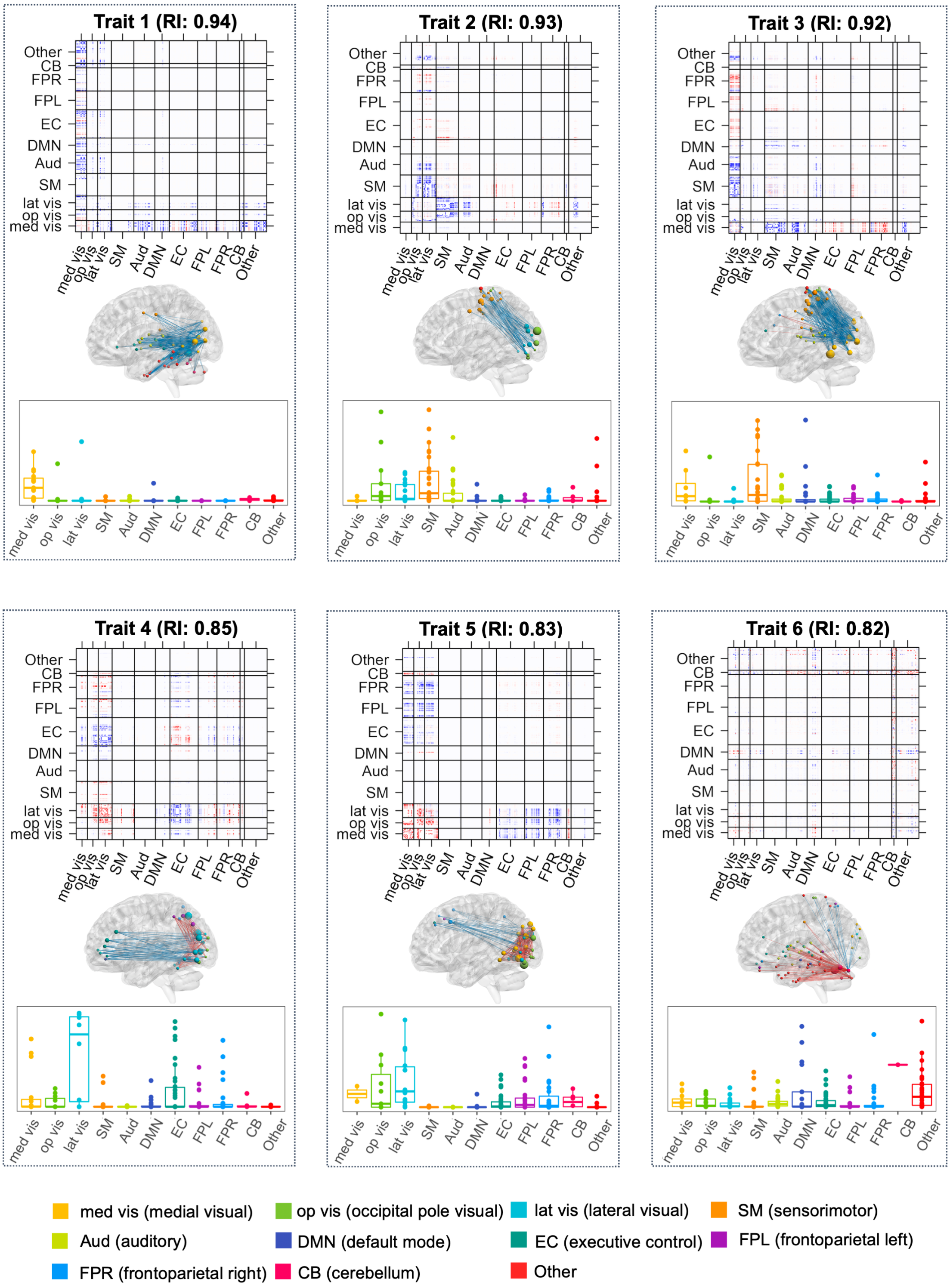


**Figure S1**: Part I: Thirty connectivity traits extracted from the PNC study ordered by their reliability index. The top 0.5% brain connections and top contributing nodes are displayed in the brain maps. Node contribution indices that help identify key brain nodes and networks driving each connectivity trait are shown in the boxplot arranged by networks.

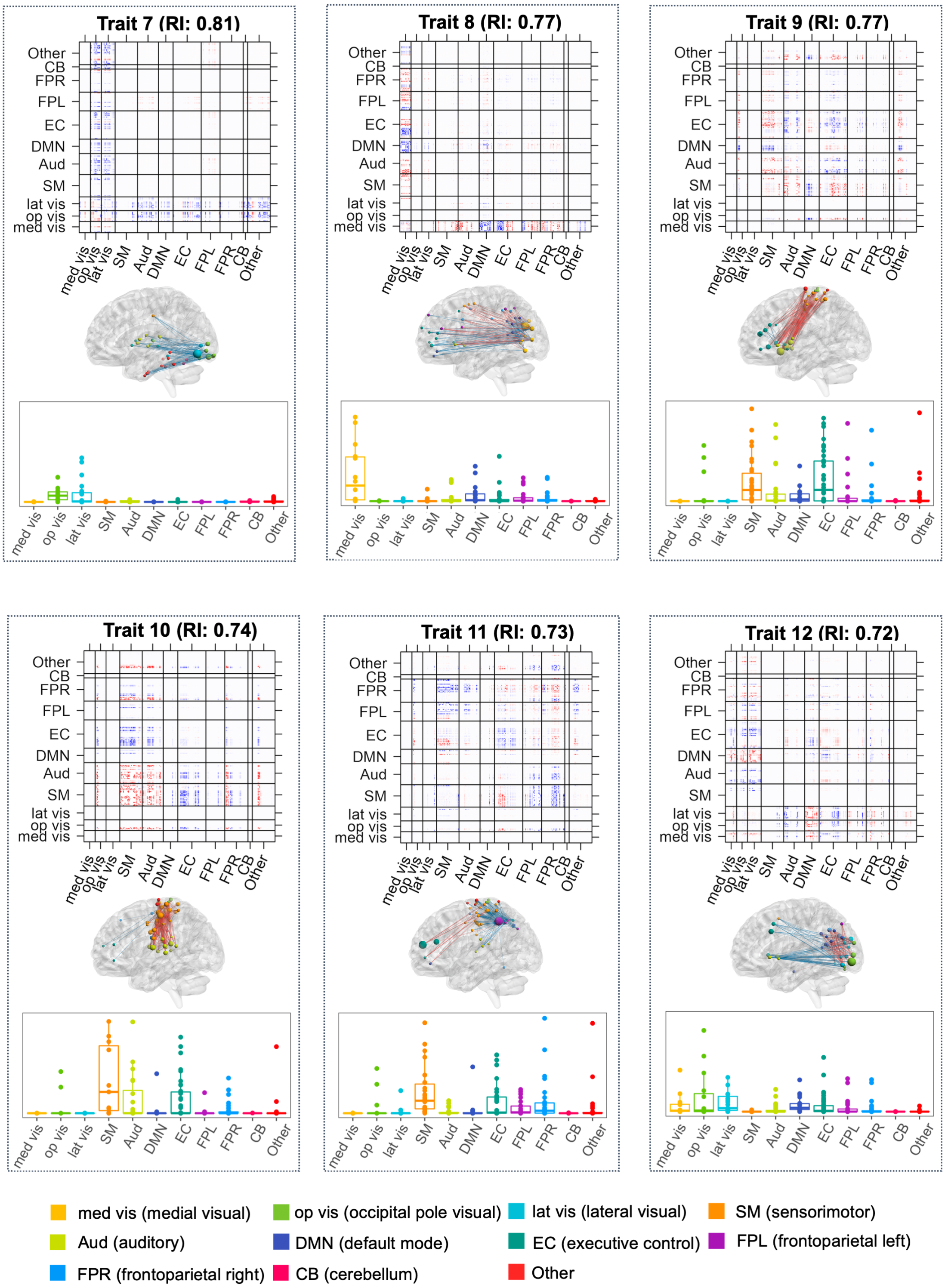


**Figure S1**: Part II: Thirty connectivity traits extracted from the PNC study ordered by their reliability index. The top 0.5% brain connections and top contributing nodes are displayed in the brain maps. Node contribution indices that help identify key brain nodes and networks driving each connectivity trait are shown in the boxplot arranged by networks.

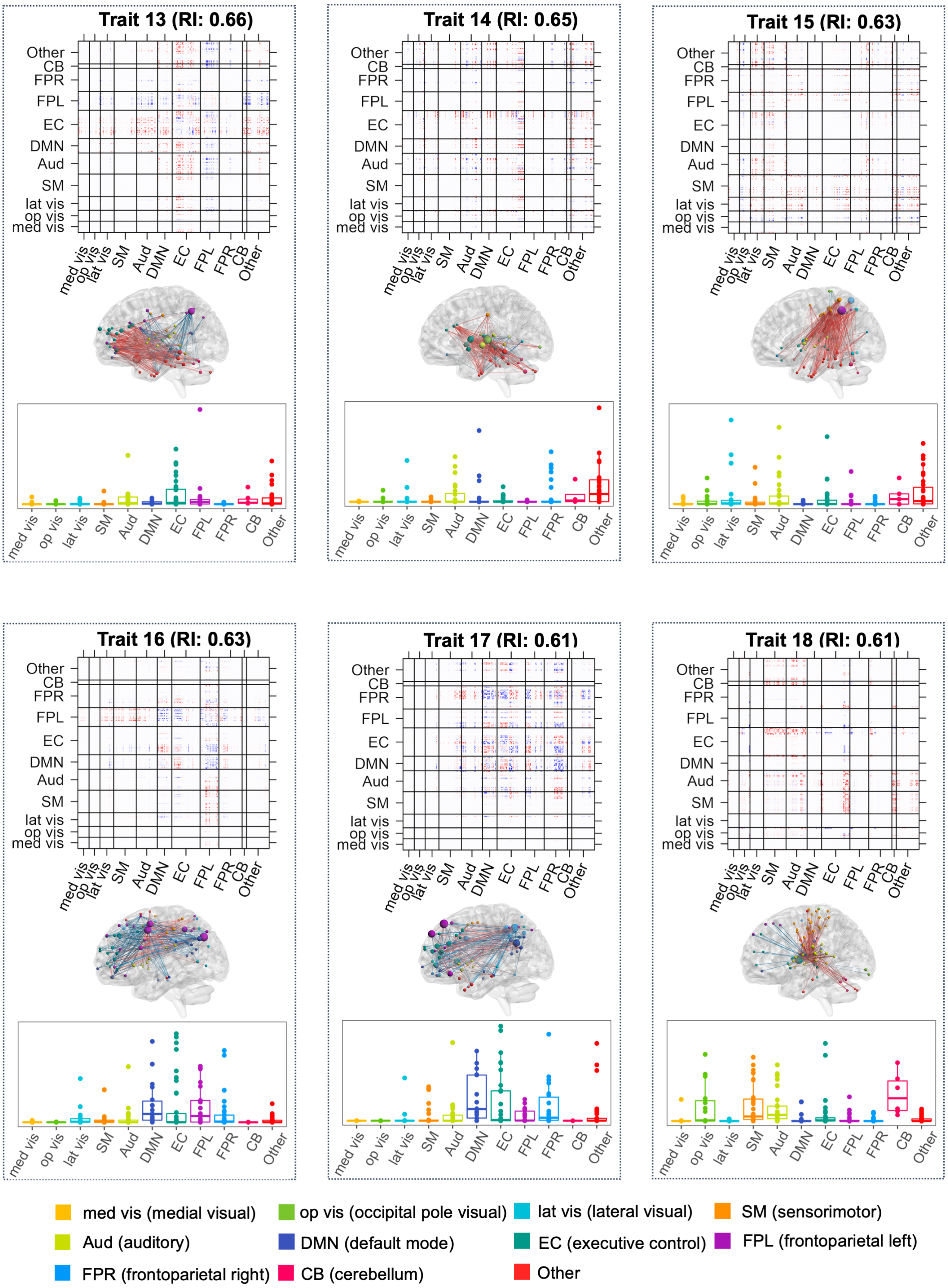


**Figure S1**: Part III: Thirty connectivity traits extracted from the PNC study ordered by their reliability index. The top 0.5% brain connections and top contributing nodes are displayed in the brain maps. Node contribution indices that help identify key brain nodes and networks driving each connectivity trait are shown in the boxplot arranged by networks.

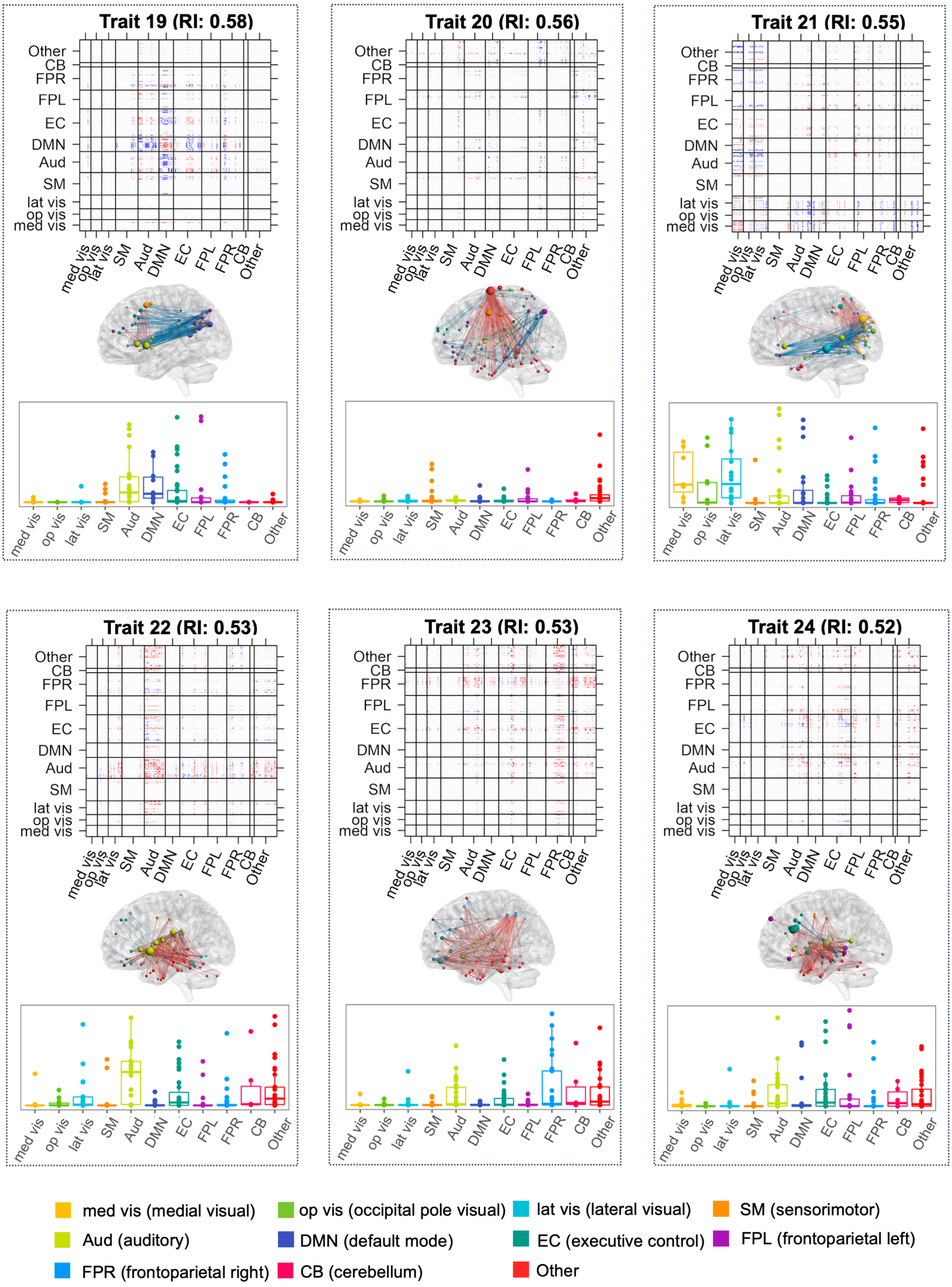


**Figure S1**: Part IV: Thirty connectivity traits extracted from the PNC study ordered by their reliability index. The top 0.5% brain connections and top contributing nodes are displayed in the brain maps. Node contribution indices that help identify key brain nodes and networks driving each connectivity trait are shown in the boxplot arranged by networks.

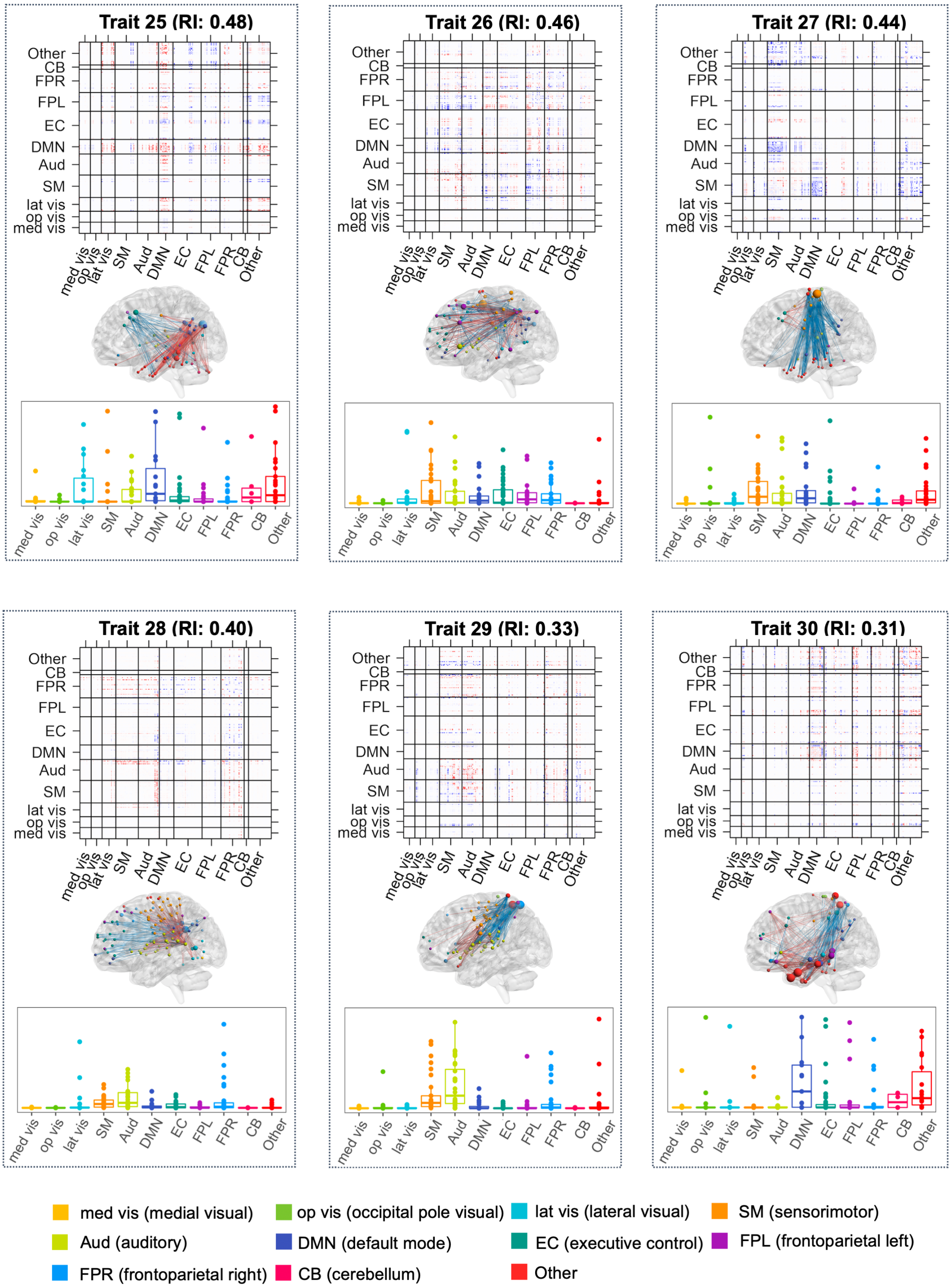


**Figure S1**: Part V: Thirty connectivity traits extracted from the PNC study ordered by their reliability index. The top 0.5% brain connections and top contributing nodes are displayed in the brain maps. Node contribution indices that help identify key brain nodes and networks driving each connectivity trait are shown in the boxplot arranged by networks.

## 2. Latent dynamic connectivity traits extracted from the PNC study using existing methods

In this section, we present the 30 dynamic latent connectivity traits uncovered by connICA (Amico et al., 2017) (Figure S2) and dictionary learning (DL) (Figure S3) by decomposing dynamic functional connectivity (dFC) measures derived from resting-state fMRI (rs-fMRI) data from the Philadelphia Neurodevelopmental Cohort (PNC) project. For each method, the traits are matched one-to-one with dyna-LOCUS traits presented in Supplementary Materials Section 1 and ordered accordingly.

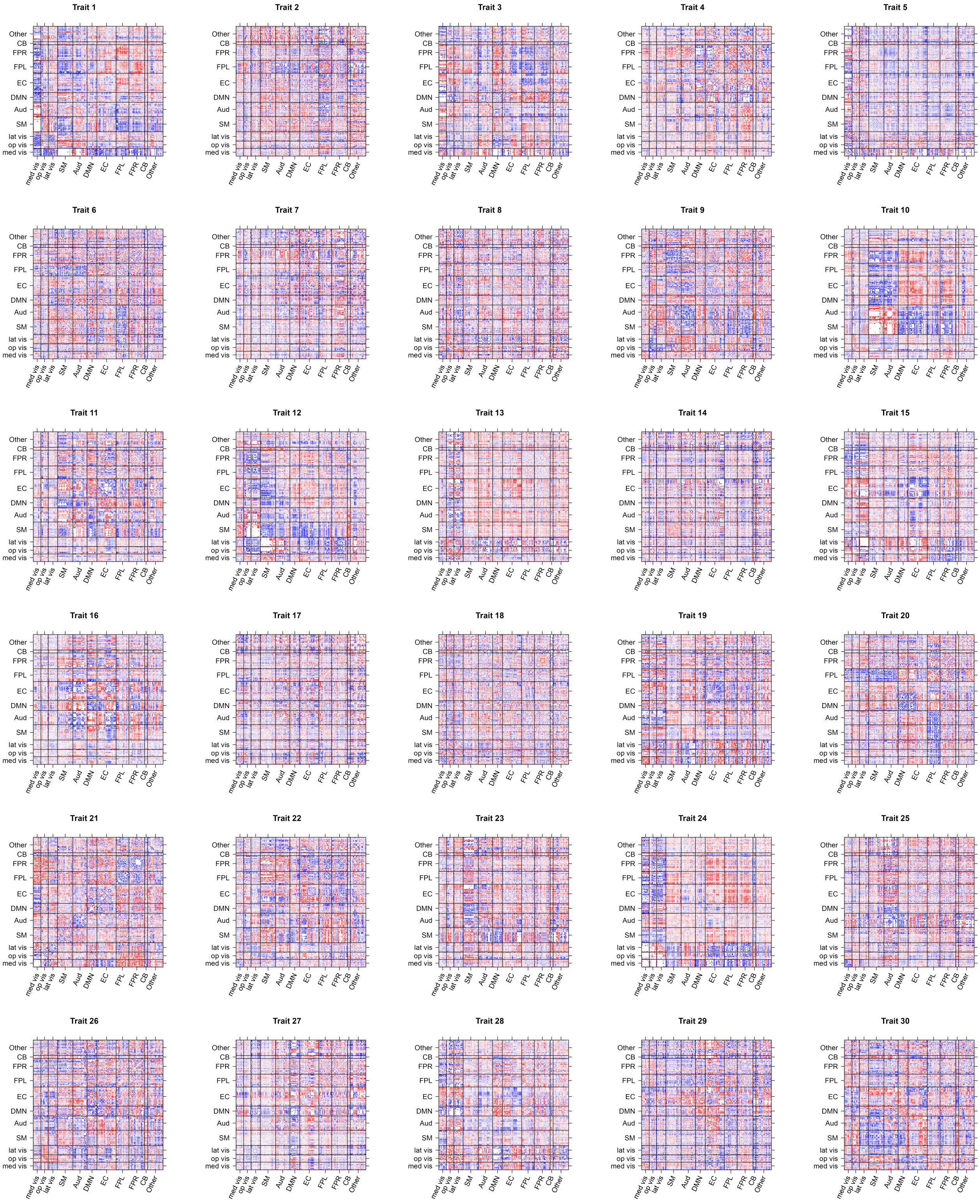


**Figure S2**: 30 dynamic latent connectivity traits uncovered by connICA using dFC measures derived from rs-fMRI data from the PNC project. These traits are matched one-to-one with the traits extracted by dyna-LOCUS and ordered accordingly.

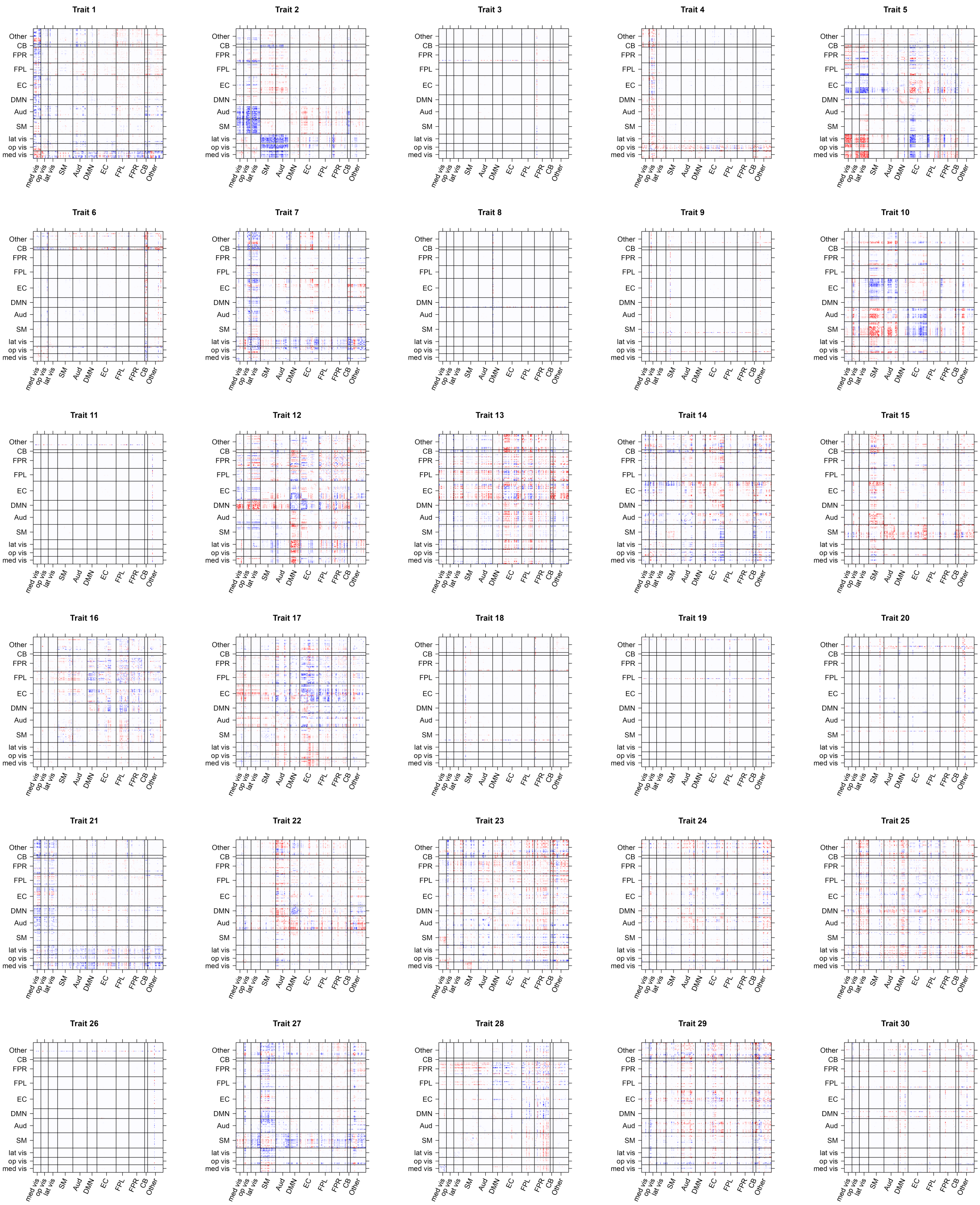


**Figure S3**: 30 dynamic latent connectivity traits uncovered by dictionary learning (DL) using dFC measures derived from rs-fMRI data from the PNC project. These traits are matched one-to-one with the traits extracted by dyna-LOCUS and ordered accordingly.

## 3. Gender and age differences in the connectivity traits

In this section, we first validate dyna-LOCUS's finding of the age and gender effect for the executive function related connectivity traits extracted from the PNC study. Among the 30 extracted traits, Trait 14 (EC-Aud-DMN-FPR) consists of connections within the executive control (EC) network and connections between the EC network and several other networks. The regression analysis of the logarithm of energy of individual's trait loadings on Trait 14 shows a significant interaction effect between age and gender. To validate this finding from dyna-LOCUS, Specifically, we generate 100 replication data samples, each consisting of a subset of 376 participants (75% of the full sample size) randomly sampled from the PNC data. We then perform the same regression analysis and test for the age and gender interaction effect in each of the replication samples. Figure S4 shows the p-values across the 100 replication samples. When there isn't age by gender interaction effect, we expect the p-value would approximately follow a uniform distribution between 0 and 1 (Moore et al., 2018), meaning they should be evenly distributed within this range. The violin plot reveals that the p-values are predominantly smaller than expected under the null hypothesis. In approximately 40% of the replication samples, the results are significant with p-values less than 0.05. And the results in approximately 60% of the replication samples are significant at the significance level of 0.1. This indicates the presence of an interaction effect between age and gender for Trait 14, validating the finding reported in the paper.

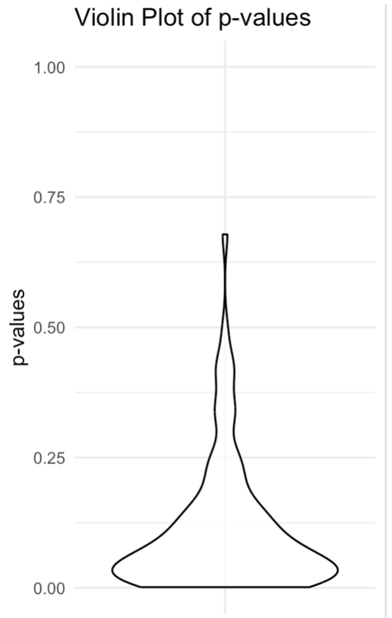


**Figure S4**: Validation of the age-by-gender interaction effect the executive function related connectivity trait (Trait 14 EC-Aud-DMN-FPR) using the data resampling method. The violin plot displays the p-values of this effect in 100 replication data samples from the PNC study.

Furthermore, we present gender and age effects for all 30 connectivity traits extracted from the PNC study by dyna-LOCUS and the existing connICA and dictionary learning (DL) methods. We match the traits from connICA and DL to those from dyna-LOCUS. We then employ regression models to model the logarithm of energy of an individual's trait loadings in terms of age, gender, and their interaction. The p-values

for these effects from each method are presented in Table S1. Due to the simultaneous hypothesis testing across all 30 traits, we implement multiple comparison corrections using the false discovery rate (FDR) method. Effects that are significant after controlling for the FDR at the 0.05 level are marked with asterisks in the table.

| Trait | dyna-LOCUS | | | connICA | | | DL | | |
|---|---|---|---|---|---|---|---|---|---|
| | gender | age | interaction | gender | age | interaction | gender | age | interaction |
| 1 | 0.466 | 0.240 | 0.889 | 0.055 | 0.609 | 0.801 | 0.378 | 0.446 | 0.642 |
| 2 | 0.921 | 0.682 | 0.369 | 0.517 | 0.023 | 0.498 | 0.658 | 0.162 | 0.280 |
| 3 | 0.938 | 0.159 | 0.296 | 0.489 | 0.203 | 0.577 | 0.089 | 0.010* | 0.356 |
| 4 | 0.846 | 0.400 | 0.928 | 0.794 | 0.887 | 0.712 | 0.909 | 0.052 | 0.053 |
| 5 | 0.819 | 0.008* | 0.526 | 0.437 | 0.051 | 0.463 | 0.831 | 0.060 | 0.218 |
| 6 | 0.333 | 0.063 | 0.175 | 0.414 | 0.208 | 0.617 | 0.326 | 0.293 | 0.169 |
| 7 | 0.794 | 0.887 | 0.712 | 0.003* | 0.230 | 0.026 | 0.322 | 0.156 | 0.295 |
| 8 | 0.437 | 0.051 | 0.463 | 0.576 | 0.005* | 0.586 | 0.031 | 0.000* | 0.042 |
| 9 | 0.697 | 0.226 | 0.267 | 0.456 | 0.382 | 0.359 | 0.892 | 0.004* | 0.933 |
| 10 | 0.055 | 0.609 | 0.801 | 0.938 | 0.159 | 0.296 | 0.503 | 0.066 | 0.706 |
| 11 | 0.104 | 0.233 | 0.276 | 0.823 | 0.574 | 0.517 | 0.780 | 0.003* | 0.058 |
| 12 | 0.280 | 0.488 | 0.384 | 0.191 | 0.797 | 0.479 | 0.044 | 0.721 | 0.309 |
| 13 | 0.003* | 0.230 | 0.026 | 0.090 | 0.002* | 0.324 | 0.212 | 0.085 | 0.862 |
| 14 | 0.493 | 0.000* | 0.030 | 0.466 | 0.240 | 0.889 | 0.146 | 0.000* | 0.018 |
| 15 | 0.000* | 0.531 | 0.598 | 0.545 | 0.023 | 0.210 | 0.132 | 0.434 | 0.886 |
| 16 | 0.130 | 0.677 | 0.458 | 0.036 | 0.001* | 0.781 | 0.004 | 0.279 | 0.672 |
| 17 | 0.191 | 0.797 | 0.479 | 0.000* | 0.531 | 0.598 | 0.834 | 0.000* | 0.085 |
| 18 | 0.517 | 0.023 | 0.498 | 0.130 | 0.677 | 0.458 | 0.264 | 0.013* | 0.252 |
| 19 | 0.036 | 0.001* | 0.781 | 0.333 | 0.063 | 0.175 | 0.041 | 0.121 | 0.243 |
| 20 | 0.024 | 0.308 | 0.004 | 0.024 | 0.308 | 0.004 | 0.008 | 0.014* | 0.056 |
| 21 | 0.456 | 0.382 | 0.359 | 0.394 | 0.171 | 0.261 | 0.052 | 0.079 | 0.102 |
| 22 | 0.545 | 0.023 | 0.210 | 0.697 | 0.226 | 0.267 | 0.075 | 0.360 | 0.976 |
| 23 | 0.090 | 0.002* | 0.324 | 0.104 | 0.233 | 0.276 | 0.474 | 0.318 | 0.398 |
| 24 | 0.489 | 0.203 | 0.577 | 0.921 | 0.682 | 0.369 | 0.975 | 0.131 | 0.253 |
| 25 | 0.166 | 0.074 | 0.407 | 0.819 | 0.008* | 0.526 | 0.054 | 0.027 | 0.732 |
| 26 | 0.576 | 0.005* | 0.586 | 0.493 | 0.000* | 0.030 | 0.121 | 0.001* | 0.025 |
| 27 | 0.823 | 0.574 | 0.517 | 0.280 | 0.488 | 0.384 | 0.423 | 0.471 | 0.564 |
| 28 | 0.257 | 0.008* | 0.007 | 0.257 | 0.008* | 0.007 | 0.646 | 0.381 | 0.925 |
| 29 | 0.394 | 0.171 | 0.261 | 0.166 | 0.074 | 0.407 | 0.552 | 0.343 | 0.452 |
| 30 | 0.414 | 0.208 | 0.617 | 0.846 | 0.400 | 0.928 | 0.793 | 0.019 | 0.061 |

**Table S1**: Age and Gender effects for all 30 connectivity traits extracted from the PNC study by dyna-LOCUS and the existing connICA and dictionary learning (DL) methods. Effects that are significant after controlling for the false discovery rate (FDR) at the 0.05 level are marked with asterisks in the table.

## 4. Additional simulation studies with varying levels of sparsity in the source signals

In this section, we evaluate the performance of dyna-LOCUS across varying sparsity levels of source signals. In addition to the simulation scenario presented in the main manuscript where the sources have a high sparsity level, we consider two additional settings where the sources exhibit decreasing levels of sparsity as the number of connections increases. Table S2 and Figure S5 depict simulation results for a medium sparsity level, while Table S3 and Figure S6 present results for a low sparsity level. Overall, dyna-LOCUS exhibits better accuracy in recovering the underlying source signals and their respective temporal loadings compared to the connICA and sparse dictionary learning (DL) methods. Results from these two additional simulation settings are consistent with the findings reported in the main manuscript for the high sparsity level setting.

| Term | N | Var. | **dyna-LOCUS** | connICA | DL |
|---|---|---|---|---|---|
| Latent Source Corr. (SD) | 20 | Low | 0.936 (0.006) | 0.801 (0.023) | 0.908 (0.011) |
| | | Mid | 0.911 (0.011) | 0.762 (0.014) | 0.890 (0.007) |
| | | High | 0.829 (0.011) | 0.675 (0.015) | 0.783 (0.020) |
| | 50 | Low | 0.939 (0.002) | 0.788 (0.001) | 0.929 (0.004) |
| | | Mid | 0.925 (0.006) | 0.771 (0.005) | 0.916 (0.002) |
| | | High | 0.901 (0.008) | 0.734 (0.010) | 0.887 (0.003) |
| Loading Matrix Corr. (SD) | 20 | Low | 0.987 (0.005) | 0.799 (0.014) | 0.917 (0.017) |
| | | Mid | 0.959 (0.005) | 0.782 (0.010) | 0.930 (0.007) |
| | | High | 0.889 (0.006) | 0.730 (0.012) | 0.857 (0.024) |
| | 50 | Low | 0.988 (0.003) | 0.792 (0.002) | 0.940 (0.003) |
| | | Mid | 0.960 (0.005) | 0.772 (0.007) | 0.922 (0.002) |
| | | High | 0.911 (0.006) | 0.731 (0.011) | 0.881 (0.003) |

**Table S2**: Simulation results for comparing dyna-LOCUS with connICA and DL for source signals with a medium sparsity level, based on 100 simulation runs conducted under three variance (Var.) settings. Values presented are mean and standard deviation of correlations between the true and estimated latent sources and loading/mixing matrices.

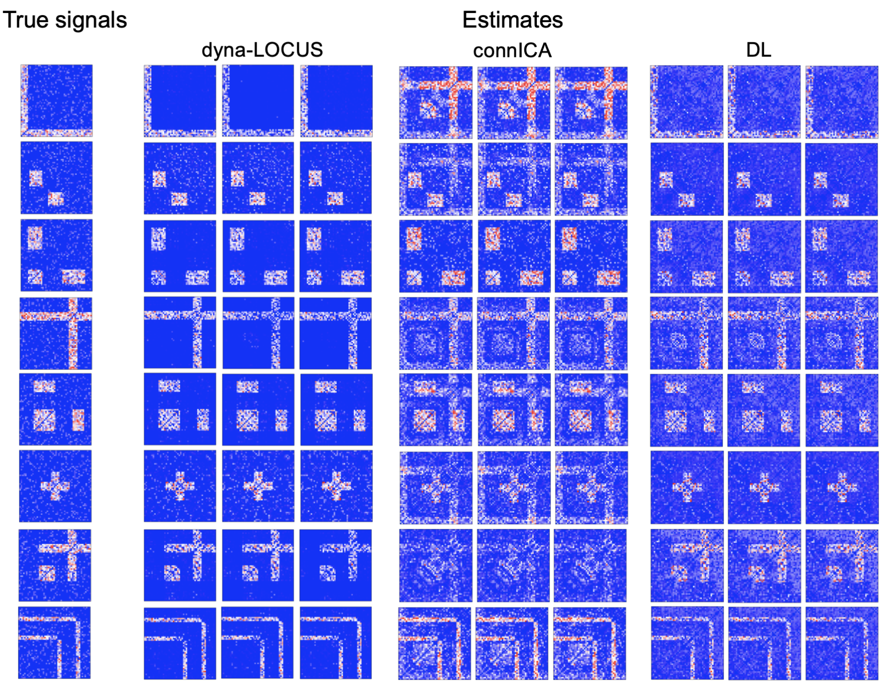


**Figure S5**: Results for the simulation study where the source signals exhibit a medium sparsity level. Figures depict the true source signals and the estimated signals by dyna-LOCUS, connICA, and DL in three randomly selected simulation runs.

| Term | N | Var. | **dyna-LOCUS** | connICA | DL |
|---|---|---|---|---|---|
| Latent Source Corr. (SD) | 20 | Low | 0.956 (0.002) | 0.806 (0.017) | 0.842 (0.029) |
| | | Mid | 0.911 (0.005) | 0.765 (0.020) | 0.893 (0.012) |
| | | High | 0.809 (0.012) | 0.688 (0.020) | 0.786 (0.024) |
| | 50 | Low | 0.958 (0.001) | 0.787 (0.005) | 0.903 (0.006) |
| | | Mid | 0.937 (0.004) | 0.772 (0.006) | 0.913 (0.007) |
| | | High | 0.885 (0.012) | 0.742 (0.003) | 0.883 (0.014) |
| Loading Matrix Corr. (SD) | 20 | Low | 0.996 (0.002) | 0.802 (0.011) | 0.834 (0.029) |
| | | Mid | 0.966 (0.004) | 0.783 (0.013) | 0.931 (0.013) |
| | | High | 0.893 (0.011) | 0.741 (0.016) | 0.857 (0.032) |
| | 50 | Low | 0.997 (0.000) | 0.791 (0.007) | 0.922 (0.004) |
| | | Mid | 0.969 (0.004) | 0.775 (0.008) | 0.916 (0.007) |
| | | High | 0.914 (0.010) | 0.742 (0.003) | 0.883 (0.013) |

**Table S3**: Simulation results for comparing dyna-LOCUS with connICA and DL for source signals with a low sparsity level, based on 100 simulation runs conducted under three variance (Var.) settings. Values presented are mean and standard deviation of correlations between the true and estimated latent sources and loading/mixing matrices.

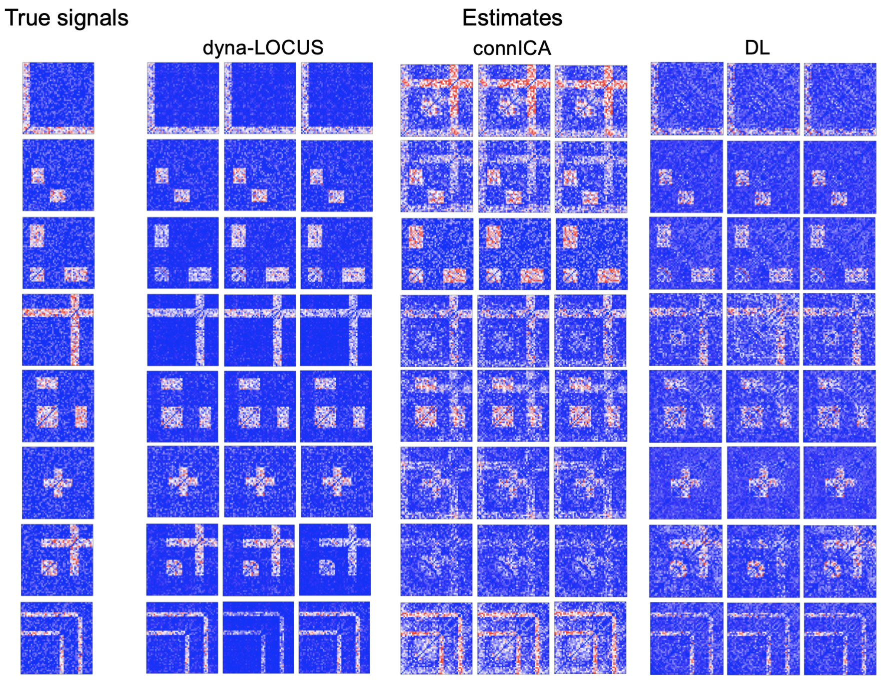


**Figure S6**: Results for the simulation study where the source signals exhibit a low sparsity level. Figures depict the true source signals and the estimated signals by dyna-LOCUS, connICA and DL in three randomly selected simulation runs.

## 5. Additional simulation study using source signals derived from the PNC study

In this section, we simulate dFC data using true source signals derived from connectivity traits extracted from the PNC study. We compare the performance of dyna-LOCUS with two other source separation methods: connICA (Amico et al., 2017) which is a recently developed connectivity ICA method, and the dictionary learning (DL) method (Mairal et al., 2009) which is a popular sparse decomposition method with $l_1$ sparsity penalization.

We specify $V = 264, q = 3$, and consider two sample sizes $N = 20, 50$ and $T = 26$ windows. We generate three latent connectivity source signals based on the connectivity traits derived directly from the PNC study (Figure S7). The mixing coefficients are also sampled from the estimates from the PNC study. Furthermore, we add zero mean Gaussian noises to the mixture of signals where the variance is specified based on signal-to-noise ratio observed from PNC data. Specifically, we consider three variance settings with $\sigma^2 = 2^2, 3^2$, and $4^2$, corresponding to low, medium, and high variance levels, respectively. In summary, we have $2 \times 3$ simulation settings with different combinations of sample sizes and variance levels. For each setting, we generate $100$ simulation runs.

Following previous work (Beckmann et al., 2005; Wang & Guo, 2019, 2023), we evaluate the performance of each method based on the correlations between the truth and the model-based estimates. We further examine the standard deviation of the correlations across $100$ simulation runs to evaluate the stability of the results.

| Term | N | Var. | **dyna-LOCUS** | connICA | DL |
|---|---|---|---|---|---|
| Latent Source Corr. (SD) | 20 | Low | 0.981 (0.000) | 0.844 (0.001) | 0.918 (0.001) |
| | | Mid | 0.925 (0.001) | 0.722 (0.001) | 0.824 (0.002) |
| | | High | 0.829 (0.002) | 0.610 (0.002) | 0.755 (0.003) |
| | 50 | Low | 0.994 (0.000) | 0.927 (0.000) | 0.969 (0.000) |
| | | Mid | 0.983 (0.000) | 0.854 (0.001) | 0.955 (0.001) |
| | | High | 0.949 (0.001) | 0.774 (0.001) | 0.924 (0.001) |
| Loading Matrix Corr. (SD) | 20 | Low | 0.996 (0.000) | 0.996 (0.000) | 0.996 (0.000) |
| | | Mid | 0.988 (0.001) | 0.987 (0.000) | 0.990 (0.000) |
| | | High | 0.970 (0.001) | 0.969 (0.001) | 0.979 (0.001) |
| | 50 | Low | 0.997 (0.000) | 0.996 (0.000) | 0.997 (0.000) |
| | | Mid | 0.991 (0.000) | 0.991 (0.000) | 0.993 (0.000) |
| | | High | 0.981 (0.000) | 0.980 (0.000) | 0.986 (0.000) |

**Table S4**: Simulation results for comparing dyna-LOCUS with connICA and DL based on 100 simulation runs conducted under three variance (Var.) levels. Values presented are mean and standard deviation of correlations between the true and estimated latent sources and loading/mixing matrices.

Results in Table S4 show that while the three methods show comparable accuracy for estimating the mixing coefficients, dyna-LOCUS consistently yields more accurate results in uncovering the latent connectivity sources. Figure S7 illustrates the true source signals alongside the estimated signals generated by dyna-LOCUS, connICA, and DL. In comparison with the two existing methods, dyna-LOCUS generates more accurate results with fewer false positive findings. Specifically, connICA, being a decomposition method without sparsity constraints and the low-rank structure, tends to yield noisy and inaccurate estimates. As a sparse decomposition method, DL produces sparse estimates for the source signals. However, it doesn't model the source signals using the low-rank structure and disregards the interdependence among brain connections. Instead, it treats connections as independent parameters, leading to a large number of parameters for DL to learn. As a result, DL yields less accurate results compared to the proposed dyna-LOCUS. For instance, for the second source signal, dyna-LOCUS successfully recovers the connections between the Visual Networks and EC, FPL, and FPR networks, while DL produces very weak or no signals for these connections and generates false positive findings between lat vis and med vis networks. Similarly, dyna-LOCUS successfully recovers the connections between SM and DMN, EC and FPR for the first source signals and the connections between the visual networks and FPL and FPR in the third source signals, while DL fails to achieve the same level of recovery.

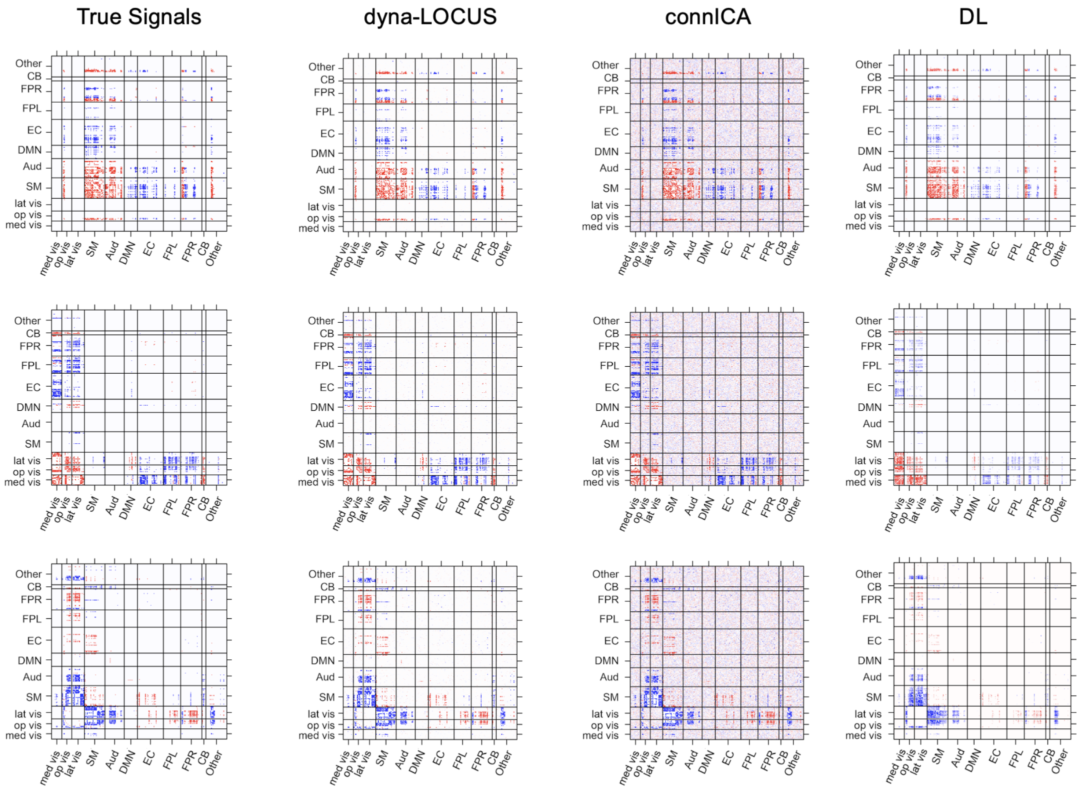


**Figure S7**: Results from the simulation study with source signals derived from the PNC study. The figures illustrate the true source signals and the estimated signals by dyna-LOCUS, connICA, and dictionary learning(DL) in three randomly selected simulation runs conducted under the low level variance setting.

## 6. Latent dynamic connectivity traits extracted from the PNC study using LOCUS

In this section, we present 30 static latent connectivity traits uncovered by LOCUS when decomposing static functional connectivity measures derived from resting-state fMRI (rs-fMRI) data from the PNC project (Figure S8). The traits are matched one-to-one with dyna-LOCUS traits in Supplementary Materials Section 1 and ordered accordingly.

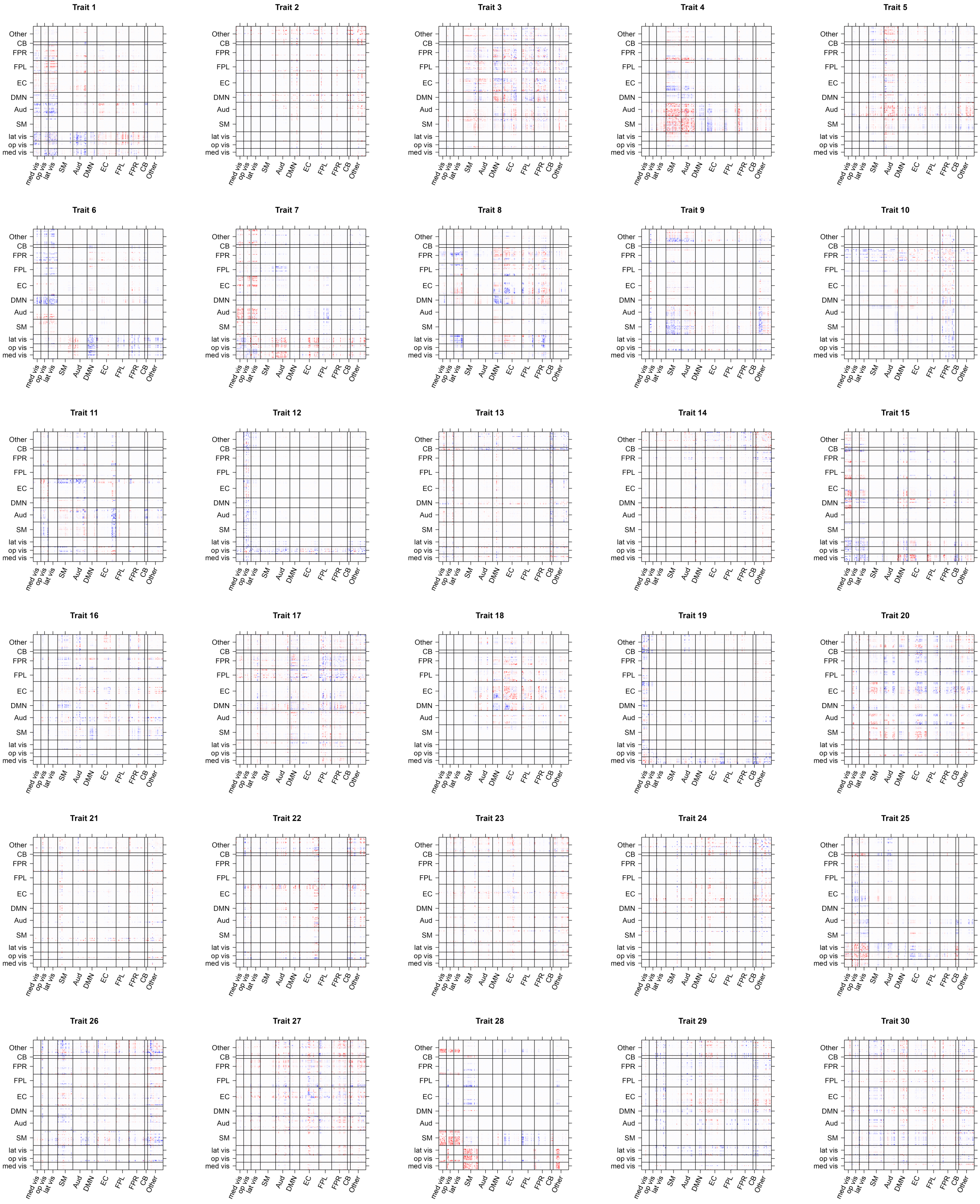


**Figure S8**: 30 static latent connectivity traits uncovered by LOCUS using static FC measures derived from rs-fMRI data from the PNC project. These traits are matched one-to-one with the dyna-LOCUS results, as detailed in Supplementary Materials Section 1.

## 7. An alternative estimation algorithm

The proposed node-rotation algorithm presented in the Algorithm 1 of Appendix B of the paper is developed based on the block multiconvexity of the objective function. It has the appealing theoretical property that updating each block of the parameters can be performed via convex optimization. Though being a highly efficient algorithm with analytic solutions, the node-rotation algorithm does involve rotating across the nodes to update the latent coordinates of each node $\boldsymbol{x}_\ell(v)(v = 1, \ldots, V)$. Here, we present an alternative estimation method to further accelerate computation in learning dyna-LOCUS, especially for studies with large sample sizes and brain atlases involving a large number of nodes. Instead of iteratively updating each node, the alternative algorithm updates the latent coordinates of all the nodes $\boldsymbol{X}_\ell$ simultaneously using an eigenvalue decomposition. Specifically, the objective function for dyna-LOCUS is:

$$\min_{\widetilde{\boldsymbol{A}},\{\boldsymbol{X}_\ell,\boldsymbol{D}_\ell\}} \sum_{l=1}^{q} ||\widetilde{\boldsymbol{Y}}^{'}\widetilde{\boldsymbol{a}}_\ell - \mathcal{L}(\boldsymbol{X}_\ell \boldsymbol{D}_\ell \boldsymbol{X}_\ell')||_F^2 + \phi \sum_{\ell=1}^{q} \sum_{u<v} |\boldsymbol{x}_\ell(u)' \boldsymbol{D}_\ell \boldsymbol{x}_\ell(v)| + \lambda ||\boldsymbol{W}\widetilde{\boldsymbol{A}}||_F^2. \quad (1)$$

We initialize the algorithm with $\hat{\widetilde{\boldsymbol{A}}}^{(0)}, \{\hat{\boldsymbol{X}}_\ell^{(0)}, \hat{\boldsymbol{D}}_\ell^{(0)}\}$ derived from estimates based on existing methods such as connICA. The algorithm then iteratively updates the parameters through the following steps: *Step 1: Updating* $\boldsymbol{X}_\ell, \boldsymbol{D}_\ell$. At the $k$th iteration, denote $\boldsymbol{b}_\ell = \mathcal{L}(\boldsymbol{X}_\ell \boldsymbol{D}_\ell \boldsymbol{X}_\ell')$. We first derive a sparse solution for $\boldsymbol{b}_\ell$ by solving the following objective function:

$$\min_{\boldsymbol{b}_\ell} ||\widetilde{\boldsymbol{Y}}^{'}\widetilde{\boldsymbol{a}}_\ell - \boldsymbol{b}_\ell||_2^2 + \phi||\boldsymbol{b}_\ell||_1 \quad (2)$$

An analytical solution $\hat{\boldsymbol{b}}_\ell$ can be obtained (Fan & Li, 2001). We map it back to the connectivity matrix form using $\mathcal{L}^{-1}$, and then conduct eigenvalue decomposition on the matrix $\mathcal{L}^{-1}(\hat{\boldsymbol{b}}_\ell)$ to obtain $\{\hat{\boldsymbol{X}}_\ell^{(k)}, \hat{\boldsymbol{D}}_\ell^{(k)}\}$. *Step 2: Updating* $\widetilde{\boldsymbol{A}}$. We update the mixing matrix $\hat{\widetilde{\boldsymbol{A}}}^{(k)}$ based on the estimates of $\{\hat{\boldsymbol{X}}_\ell^{(k)}, \hat{\boldsymbol{D}}_\ell^{(k)}\}$, following the same procedure as the node-rotation algorithm.

We compare the results of the node-rotation algorithm and the alternative estimation algorithm using 50 datasets obtained by bootstrapping the PNC data, with each dataset containing 514 subjects. Figure S9(A) presents the correlations of the connectivity traits and their temporal loadings obtained using the two algorithms across the 50 datasets. The results produced by the alternative algorithm are consistent with those from the node-rotation algorithm. Figure S9(B) shows the computation time across the 50 datasets. On average, the alternative algorithm reduces the computational time by 19.2%.

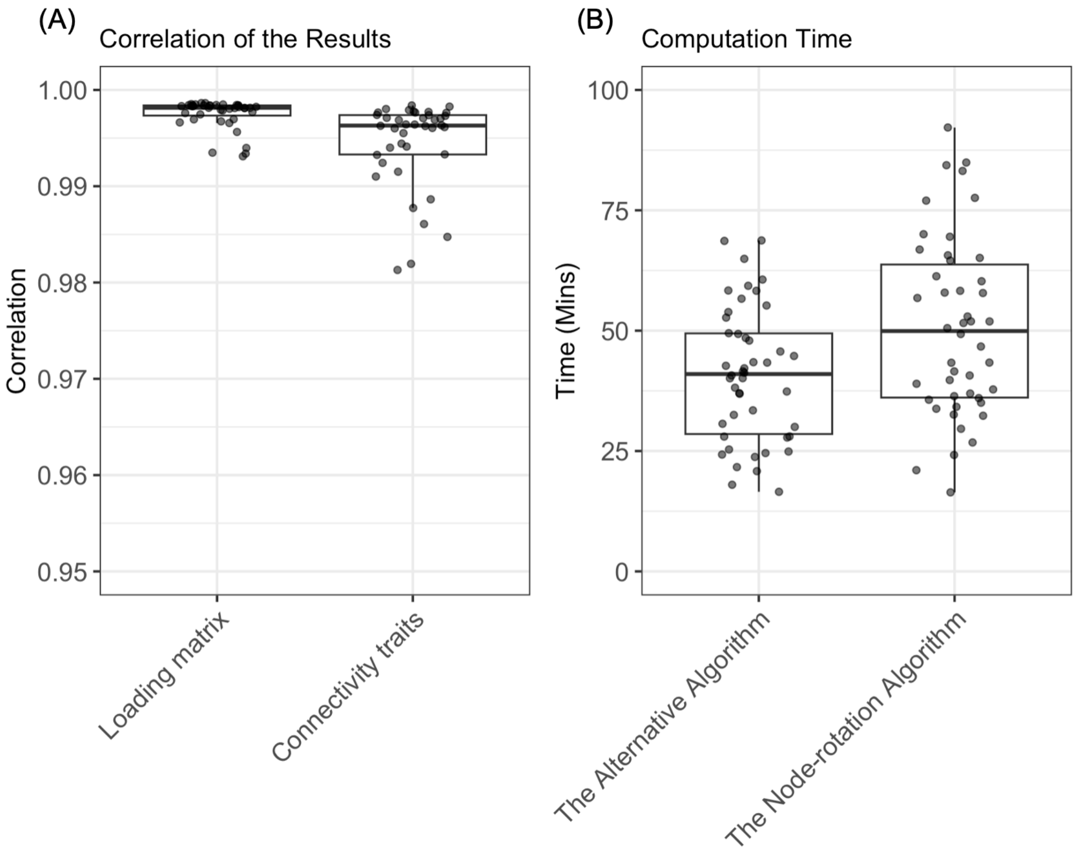


**Figure S9**: Comparison of the results and computation time between the node-rotation algorithm and the alternative algorithm based on 50 bootstrap datasets from the PNC study.

## References

Amico, E., Marinazzo, D., Di Perri, C., Heine, L., Annen, J., Martial, C., Dzemidzic, M., Kirsch, M., Bonhomme, V., Laureys, S., & Joaquín, G. (2017). Mapping the functional connectome traits of levels of consciousness. *Neuroimage*, *148*, 201–211.

Beckmann, C. F., DeLuca, M., Devlin, J. T., & Smith, S. M. (2005). Investigations into resting-state connectivity using independent component analysis. *Philosophical Transactions of the Royal Society B: Biological Sciences*, *360*(1457), 1001–1013.

Fan, J., & Li, R. (2001). Variable selection via nonconcave penalized likelihood and its oracle properties. *Journal of the American statistical Association*, *96*(456), 1348–1360.

Mairal, J., Bach, F., Ponce, J., & Sapiro, G. (2009). Online dictionary learning for sparse coding. *Proceedings of the 26th annual international conference on machine learning*, 689–696.

Moore, D. S., McCabe, G. P., & Craig, B. A. (2018). *Introduction to the practice of statistics*. W. H. Freeman.

Wang, Y., & Guo, Y. (2019). A hierarchical independent component analysis model for longitudinal neuroimaging studies. *NeuroImage*, *189*, 380–400.

Wang, Y., & Guo, Y. (2023). Locus: A regularized blind source separation method with low-rank structure for investigating brain connectivity. *The Annals of Applied Statistics*, *17*(2), 1307–1332.
